# Regulation of meristem and hormone function revealed through analysis of directly-regulated SHOOT MERISTEMLESS target genes

**DOI:** 10.1038/s41598-024-83985-1

**Published:** 2025-01-02

**Authors:** Tamara Lechon, Nicholas A. Kent, James A. H. Murray, Simon Scofield

**Affiliations:** School of Biosciences, Cardiff University, Cardiff, CF10 3AX UK

**Keywords:** Cell fate, Patterning, Plant morphogenesis, Plant stem cell, Shoot apical meristem, Plant molecular biology, Development, Gene expression, Plant genetics, Sequencing, Transcription

## Abstract

The Arabidopsis *Knotted1*-like homeobox (*KNOX*) gene *SHOOT MERISTEMLESS* (*STM*) encodes a homeodomain transcription factor that operates as a central component of the gene regulatory network (GRN) controlling shoot apical meristem formation and maintenance. It regulates the expression of target genes that include transcriptional regulators associated with meristem function, particularly those involved in pluripotency and cellular differentiation, as well as genes involved in hormone metabolism and signaling. Previous studies have identified KNOX-regulated genes and their associated *cis*-regulatory elements in several plant species. However, little is known about STM-DNA interactions in the regulatory regions of target genes in Arabidopsis. Here, we identify and map STM binding sites in the Arabidopsis genome using global ChIP-seq analysis to reveal potential directly-regulated STM target genes. We show that in the majority of target loci, STM binds within 1 kb upstream of the TSS, with other loci showing STM binding at more distal enhancer sites, and we reveal enrichment of DNA motifs containing a TGAC and/or TGAT core in STM-bound target gene *cis*-regulatory elements. We further demonstrate that many STM-bound genes are transcriptionally responsive to altered levels of STM activity, and show that among these, transcriptional regulators with key roles in meristem and hormone function are highly represented. Finally, we use a subset of these target genes to perform Bayesian network analysis to infer gene regulatory associations and to construct a refined GRN for STM-mediated control of meristem function.

**Supplementary Information:**

The online version contains supplementary material available at 10.1038/s41598-024-83985-1.

## Introduction

Shoot growth in higher plants is an iterative process that relies on populations of undifferentiated, pluripotent cells located in specialised stem cell-containing structures termed meristems to enable continued production of lateral organs such as leaves and flowers throughout the plant life cycle^1,2^. Cell fate decisions in the SAM are controlled by transcription factors and other regulatory proteins that operate in gene regulatory networks (GRNs) to maintain pluripotency and stem cell homeostasis via repression of cell differentiation, or to initiate lateral organ development through the repression of pluripotency and activation of organ-specific differentiation programmes in incipient lateral organ primordia^3–5^.

Class-1 *Knotted1*-like homeobox (*KNOX*) genes such as *KNOTTED1* (*KN1*) from maize and its ortholog *SHOOT MERISTEMLESS* (*STM*) from Arabidopsis encode TALE homeodomain transcription factors that are essential for conferring pluripotency to cells in the SAM^6,7^. *KNOX* genes are expressed throughout the SAM, including the central zone of stem cells and the surrounding peripheral zone of transit amplifying cells, but are transcriptionally repressed in incipient organ primordia and developing organs by multiple factors including the transcriptional repressor AS1, the LOB-domain protein AS2, members of the YABBY transcription factor family, the Polycomb Repressive Complex (PRC) and the class-2 TCP transcriptional regulators^8–17^. *STM* has previously been shown to be essential for embryonic SAM formation and continued SAM maintenance throughout the plant life-cycle, with loss-of-function mutants either failing to develop a SAM during embryogenesis, or developing defective SAMs that exhibit stem cell depletion caused by inappropriate organogenesis from the central zone of stem cells^18,19^. Conversely, ectopic expression of STM inhibits proper cellular differentiation in leaves, leading to leaf lobing and to *de novo* shoot meristem formation on the adaxial leaf surface^20–22^. This highlights the need for repression of *KNOX* gene expression in simple leaf species such as Arabidopsis, though reactivation of *KNOX* gene expression is required for leaflet formation in plant species with compound leaves^23,24^. Taken together, these observations suggest a critical role for STM in the control of pluripotency associated with SAM function.

Several studies have sought to understand the *KNOX* gene regulatory network by identifying KNOX-responsive target genes and examining the KNOX-DNA interactions at target gene promoters. For example, in maize and rice the principal target genes of the KNOX proteins KN1 and OSH1 were identified as being involved in phytohormone biosynthesis, signalling and response, especially relating to auxin and brassinosteroids^25–27^. In Arabidopsis and barley, *STM* or its ortholog *BKn3* have also been implicated in the regulation of auxin biosynthesis, transport and response, with several auxin-associated factors showing differential expression following ectopic expression^28,29^. Auxin accumulation in incipient organ primordia is coincident with down-regulation of STM expression, and auxin response factors (ARFs) promote histone deacetylation leading to transcriptional repression of *STM* in incipient floral primordia^30^, suggesting antagonism between auxin and *KNOX* gene function.

*KNOX* genes have also been shown to repress the biosynthesis of gibberellic acid (GA), accumulation of which is detrimental to sustained SAM function, through direct transcriptional repression of genes encoding GA biosynthetic enzymes^31–35^. Furthermore, in Arabidopsis, STM has been shown to promote cytokinin biosynthesis by activating expression of several members of the *ISOPENTYL TRANSFERASE* (*IPT*) gene family^32,36^. Cytokinin is required for proper SAM function and promotes cell division through the Cyclin D pathway^32,36–38^. Hence, KNOX proteins impinge on several phytohormone pathways that affect cell division, cell expansion and cell differentiation processes associated with SAM development/maintenance and lateral organ formation.

In Arabidopsis, STM was shown to regulate the expression of many transcription factors associated with meristem development and the control of pluripotency^29^. These included the class-1 *KNOX* genes *KNAT1/BP* and *KNAT2*, the AP2 family gene *PLT7/AIL7*, which promotes pluripotency and the regulation of phyllotactic patterning^39–41^, *CUC1* and *BOP2*, which are associated with meristem-organ boundary establishment, regulation of *KNOX* gene expression and organ polarity^42–49^ and *HB25* which promotes shoot identity^50^. STM was also shown to repress expression of the class-2 TCP transcription factor-encoding genes *TCP3*, *TCP4* and *TCP10*, which promote cellular differentiation and themselves repress *KNOX* gene expression during leaf development and antagonise meristem function when ectopically expressed^15,51–54^, revealing a functional mechanism for the inhibition of cellular differentiation associated with leaf formation by KNOX transcriptional regulators.

In this study, we comprehensively map the STM binding sites in the Arabidopsis genome using chromatin immunoprecipitation of STM-bound DNA followed by whole genome next-generation DNA sequencing (ChIP-seq). We reveal distinct STM binding sites in target gene regulatory regions, with positions ranging from proximal promoter to distal regions several kilobases upstream or downstream of the transcriptional start site (TSS). We identify several putative STM-bound *cis*-regulatory motifs, all of which contain the TGAC or TGAT cores previously shown to be required for the binding of homeodomain proteins to DNA. We then correlate STM binding sites with genes previously identified as being transcriptionally responsive to STM, revealing direct regulation of many genes that are involved in SAM development. To investigate STM GRN topology, we perform Bayesian network analysis to infer regulatory relationships among the STM target genes that encode transcriptional regulators. Together our results provide key information on the STM-DNA interactions that are associated with the promotion of pluripotency and the repression of differentiation in the SAM of Arabidopsis.

## Results

### Genome-wide identification of putative STM-binding sites by ChIP-seq

KNOX homeodomain transcriptional regulators typically bind to *cis*-acting DNA elements containing a TGAC core motif, often located in the promoter region upstream of the target locus coding sequence^25^. Previous studies have shown that STM binds to a *cis*-regulatory element ~ 200 bp upstream from the translation initiation codon of the *CUC1* gene, which encodes a NAC domain transcription factor that is essential for activation of *STM* expression during embryogenesis and for the proper establishment of meristem-organ boundary zones^29,42^. Other STM targets such as in *BOP2* or *ATHB25* show STM binding at positions further upstream of the promoter region, while other KNOX proteins bind to *cis*-regulatory elements in the gene body, such as in the *GA20ox1* gene in maize^29,55^. Hence, STM binding elements can occur at a range of distances from the transcriptional start site, necessitating a global NGS driven ChIP-seq approach to discover STM binding sites rather than targeted ChIP-PCR analysis based on in silico predictions.

**Fig. 1:**
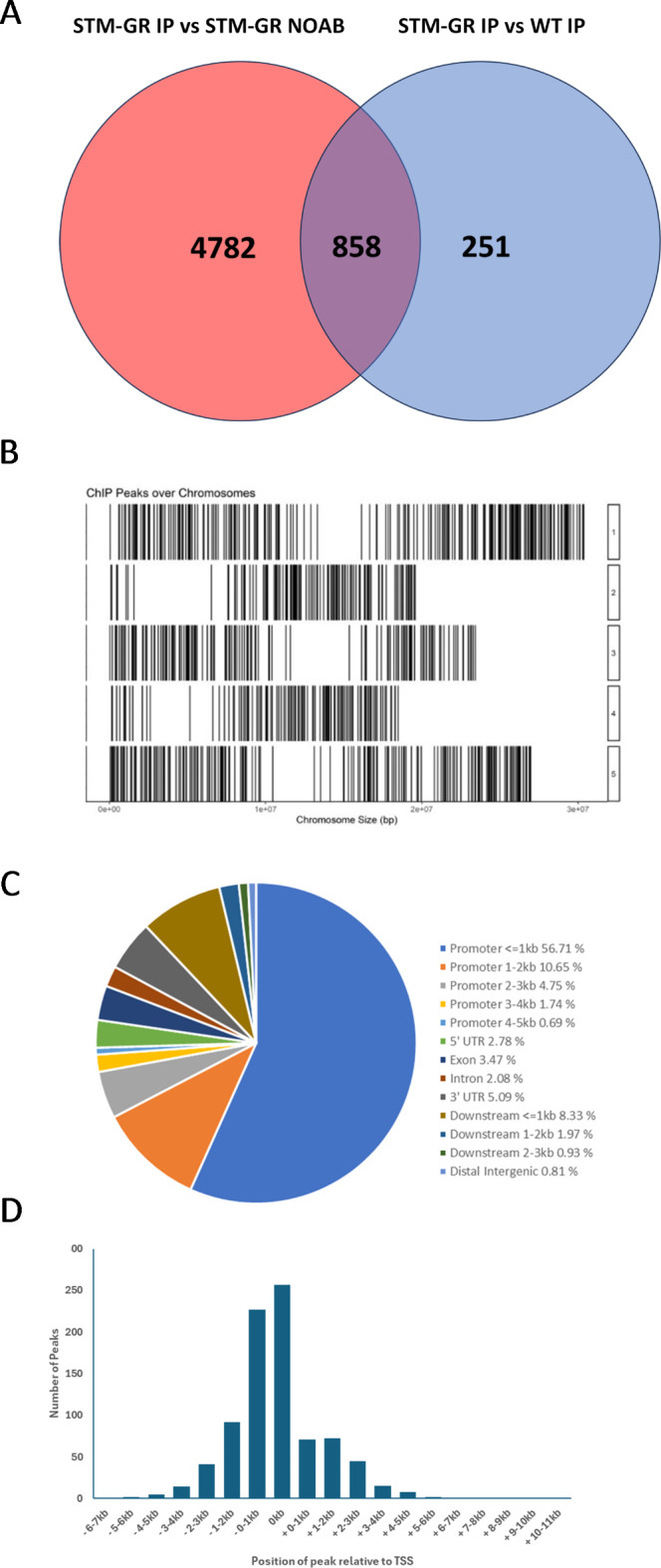
ChIP-seq analysis of putative STM-binding sites in the *Arabidopsis thaliana* genome. (**A**) Venn diagram showing the overlap between the number of ChIP-seq peaks identified by comparison of the 35S::STM-GR sample immunoprecipitated with the anti-GR antibody (STM IP) compared to the 35S::STM-GR sample mock-immunoprecipitated with no antibody (STM NoAB) and the 35S::STM-GR sample immunoprecipitated with the anti-GR antibody compared to a WT sample also immunoprecipitated with the anti-GR antibody. (**B**) Distribution of ChIP-seq peaks throughout the five chromosomes of the Arabidopsis genome. Chromosome number is shown in boxes on the righthand side, chromosome length extends from left to right. Black vertical lines indicate positions of ChIP-seq peaks. (**C**) Location of ChIP-seq peaks in relation to gene model for closest genes to ChIP-seq peaks in the Arabidopsis genome. (**D**) Histogram showing the distribution of peak positions relative to the transcriptional start site (TSS) of STM-bound genes. The full list of ChIP-seq peaks, positions and associated genes is shown in Supplementary Table S1.

To perform a global identification of the DNA binding sites for STM in the Arabidopsis genome, we performed a ChIP-seq experiment using a fusion of the *STM* coding sequence to the rat glucocorticoid receptor (GR), placed under control of the constitutive CaMV 35S promoter. CaMV 35S:*STM-GR* (*STM-GR*) plants were treated with the glucocorticoid analogue dexamethasone (DEX; allowing nuclear import of the STM-GR fusion protein) and immunoprecipitated with the anti-GR epitope antibody PA-516. We compared DEX-treated *STM-GR* anti-GR immunoprecipitated samples (STM-GR IP) with mock-immunoprecipitated no-antibody (NOAB) samples from the same DEX-treated *STM-GR* line (STM-GR NOAB), and with DEX-treated WT immunoprecipitated and mock-immunoprecipitated no-antibody samples (WT IP and WT NOAB) which served as no-GR epitope controls. By performing these two types of comparison we were better able to identify only the most robust putative STM-binding sites in the genome which were used exclusively for further analysis. This yielded a total of 858 ChIP-seq peaks, containing putative STM binding sites, distributed across all five chromosomes (Fig. 1A, B), which were then assigned to the closest genomic locus. The 858 peaks were associated with 859 loci, as some peaks were potentially associated with 2 loci, for example when the peak was located in the downstream intergenic region approximately equidistant between two loci (6 in total). Of the 859 loci, 854 had a single associated peak containing at least one putative STM-binding site, while five had two distinct peaks at different positions (Supplementary Table S1).

The ChIP-seq results show that the majority of the 858 peaks (56.7%) lie within 1 kb upstream of the transcription start site (TSS) of target loci (Fig. 1C, D). Of these, 257 peaks (~ 30%) directly overlapped the TSS. Fewer peaks (10.7%) were located between 1 kb and 2 kb upstream of the TSS, while 7.2% of peaks located between 2 kb and 5 kb upstream of the TSS. Furthermore, 13.4% of peaks were identified within the gene body (5’ and 3’ UTRs, exons and introns), while 11.2% of peaks were located downstream of target loci to up to 3 kb distal from the 3’ end of the gene body, and 0.8% were located in distal intergenic regions. These results show that the majority of putative STM-binding sites lie in the promoter region at, or upstream of, the TSS, consistent with previous analyses of KNOX proteins binding sites^25,26,29^.

### Identification of putative consensus *cis*-regulatory DNA motifs in ChIP-seq peaks

We next sought to identify putative *cis*-regulatory motifs in the 858 peaks identified in the ChIP-seq analysis. KNOTTED1-like homeodomain proteins have previously been shown to bind to *cis*-acting DNA elements containing a core TGAC motif^33,34,42,55–58^, while other homeodomain proteins such as ANTENNENAPEDIA and WUSCHEL have shown a binding preference for TAAT or TGAA^59–64^.

**Fig. 2:**
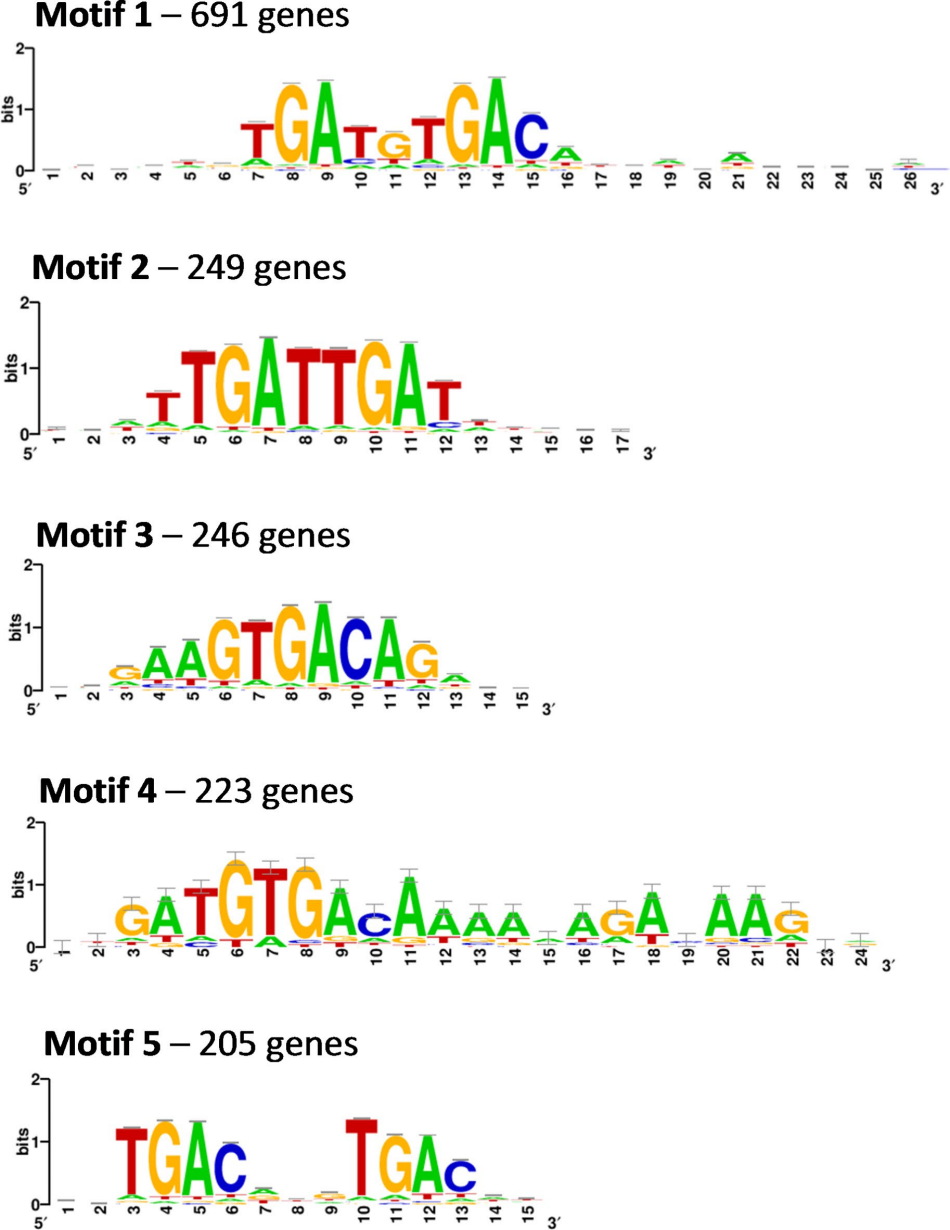
Identification of putative *cis*-regulatory motifs in STM target gene promoters. Motifs were identified by consensus DNA-binding motif analysis using a matrix-based hidden Markov model using the sequences of the 858 peaks as the input data. The number of genes containing each motif in their respective ChIP-seq peak is shown. The full list of genes associated with each motif is shown in Supplementary Table S2.

We performed a consensus DNA-binding motif analysis using a matrix-based hidden Markov model on the sequences of the 858 peaks identified by ChIP-seq analysis. This generated three distinct consensus motifs containing one or more TGAC cores, one motif containing a TGAT dyad core and one motif containing both TGAC and TGAT cores (Fig. 2; Supplementary Data 1). The latter motif was represented in the majority (691) of the 858 ChIP-seq peaks, with others being present in ~ 200–250 genes each (Supplementary Table S2). Some ChIP-seq peaks contained more than one consensus motif. We compared the abundance of these motifs with random genomic sequences from Arabidopsis and synthetic DNA sequences based on the Arabidopsis genome nucleotide composition, and found significantly higher occurrence of the motifs in the ChIP-seq peaks compared to these control sequences, suggesting that these motifs are significantly overrepresented in the ChIP-seq dataset. Overall, this strongly suggests that the core STM binding motif is similar to that of other KNOX homeodomain transcription factors. However, we could not detect any of the five motifs in some of the genes identified (58) in the ChIP-seq analysis, including well-characterised STM target genes such as *CUC1*. This was attributable to our stringent *p*-value (< 1e-5) for binding site prediction, with motifs present in the *CUC1* promoter failing to reach the required threshold for significance. Hence our approach identified a number of putative *cis*-regulatory elements but was unable to capture all biologically relevant motifs in target loci.

### Gene ontology enrichment analysis of genes containing putative STM-binding sites

**Fig. 3:**
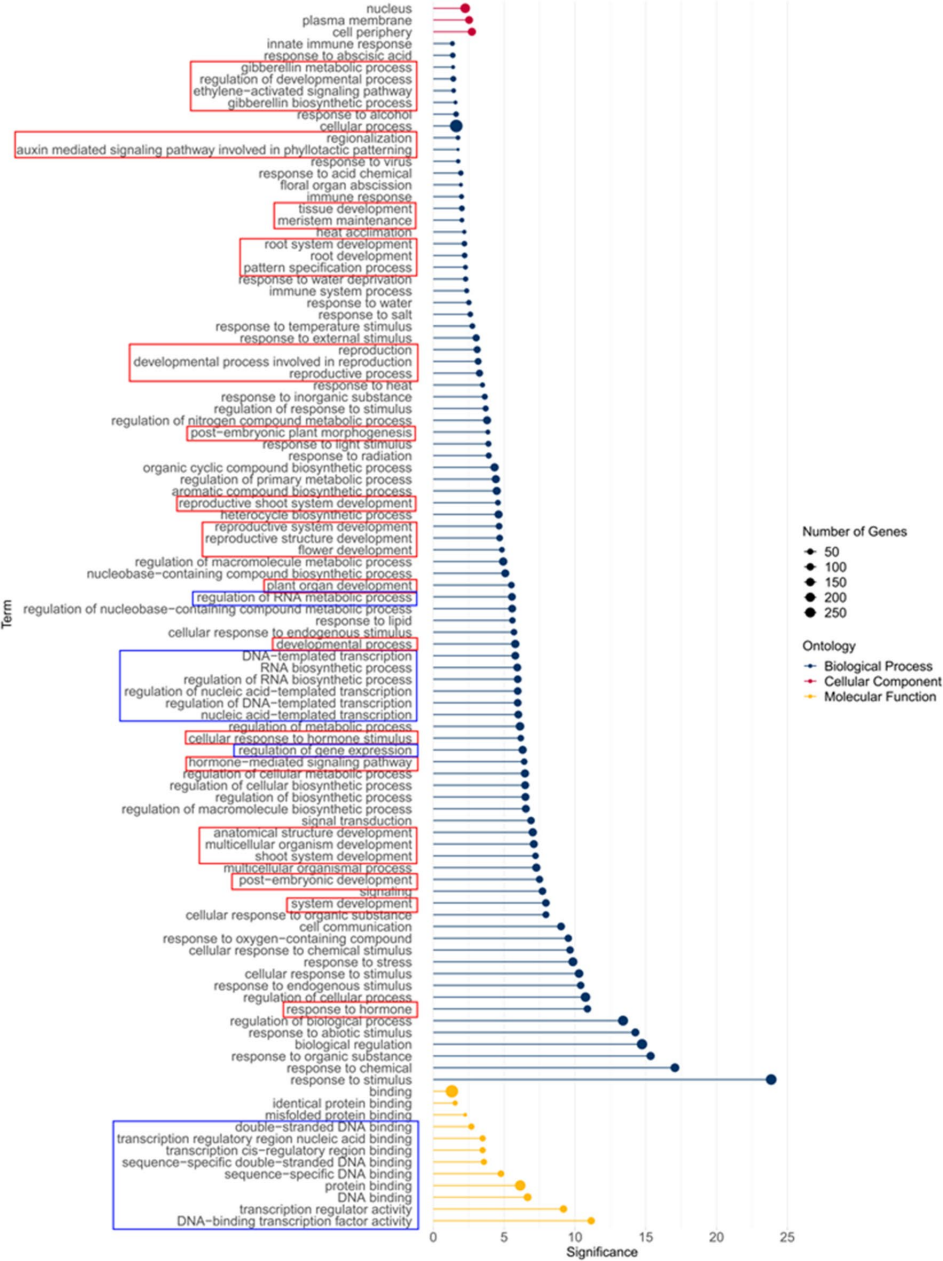
Gene Ontology (GO) Enrichment Analysis of 859 genes identified in the ChIP-seq analysis. GO terms for Cellular Component, Biological Process and Molecular Function are shown. Terms associated with development and hormones are highlighted in red boxes, while genes associated with transcription are highlighted in blue boxes. The full list of enriched GO terms is shown in Supplementary Table S3.

To determine the functions of the putative target genes identified, we performed gene ontology (GO) enrichment analysis on the 859 genes identified as having putative STM-binding sites by ChIP-seq (Fig. 3 and Supplementary Table S3). Enriched GO categories for biological process (BP) included many related to developmental processes including shoot system development, pattern specification, flower and root development, post-embryonic plant morphogenesis, hormone synthesis and hormone-mediated signalling (including auxin-mediated signaling associated with phyllotaxis). Other biological processes included transcription, cell communication and signaling, biosynthetic processes and responses to various endogenous and exogenous stimuli. Enriched molecular function (MF) categories included DNA-binding transcription factor activity and protein binding, while cellular component (CC) enrichment was shown for the nucleus, plasma membrane and cell periphery. These enriched GO categories are broadly in line with those identified in previous studies for the functions of KNOX target genes^25,26,29^, and suggest that transcriptional regulators related to development comprise a key category of STM-regulated target genes.

### Correlation of genes containing putative STM-binding sites with STM transcriptionally-responsive target genes

The data presented above show which loci are bound by STM, and include many known genes associated with meristem development (primarily transcription factors), meristem-organ boundary specification, lateral organ development, and the biosynthesis and signaling of the plant hormones auxin, cytokinin and GA (Supplementary Table S1). However, this analysis does not reveal which of these genes are also transcriptionally responsive to STM, and thus represent genuine ‘functional’ STM target genes. Indeed, several studies have shown that many TFs have a multitude of genomic binding sites that are not associated with transcriptional regulation - so-called non-functional binding sites^65–67^. We therefore compared our ChIP-seq data with published transcriptomics data that identified genes that show differential expression (i.e. are either up- or down-regulated) to altered levels of STM activity.

**Fig. 4:**
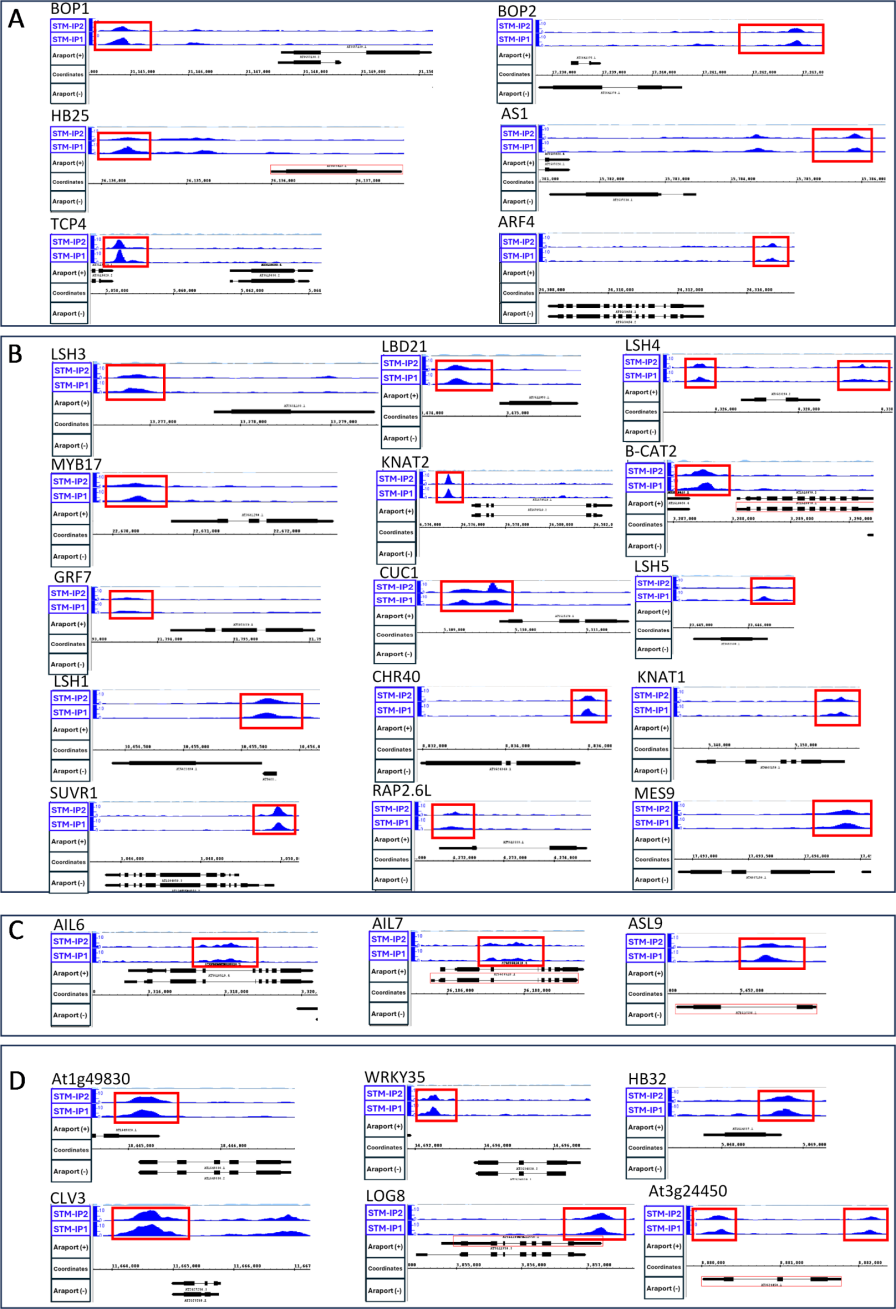
ChIP-seq peaks for selected STM target genes. (**A**) Genes with STM binding site substantially upstream of target locus TSS. (**B**) Genes with STM binding site upstream or around the target gene TSS. (**C**) Genes with STM binding site within middle of gene body. (**D**) Genes with STM binding site at distal end or downstream of target gene. The full data tracks with all controls are shown in Supplementary Figure S1.

We previously identified STM-responsive genes in a time-course of *STM* expression using the DEX-inducible TGV system followed by mRNA differential expression analysis and global meta-analysis of the differentially expressed genes in inducible STM-overexpression and RNAi experiments, and we identified putative directly-regulated STM target genes using the DEX-inducible STM-GR line induced with DEX alone or with DEX and the protein synthesis inhibitor cycloheximide, followed by differential gene expression analysis^29^. We compared the loci identified in our ChIP-seq experiment with genes previously identified in the *STM* overexpression time-course/ meta-analysis and in the direct target experiment (Supplementary Table S4). Overall, we found that 432/859 genes identified in the ChIP-seq analysis showed a transcriptional response to altered levels of STM activity, with 428 of these identified in the time-course/ meta-analysis and an additional four genes identified only in the direct-target experiment. We classified genes as ‘early-responding’ if they initially showed differential expression in the 3 h direct-target experiment, the 8 h or 24 timepoints in the timecourse or the meta-analysis (74 genes; Supplementary Table S5), while genes that were shown to be differentially expressed only in the 72 h or 9-day timepoints were classified as ‘late-responding’ (358 genes; Supplementary Table S6). At these later timepoints, the phenotypic consequences of ectopic *STM* expression, such as impaired leaf differentiation, were apparent in newly emerging leaves (72 h) or throughout all the aerial tissues (9D;^29^). The ChIP-seq peaks from the STM-IP samples for a subset of target genes are shown in Fig. 4, with additional data from control samples in Supplementary Figure S1.

We once again performed GO enrichment analysis to investigate the functions of the genes identified in the early- and late-responding datasets. For the early-responding genes, most enriched GO terms related to development and transcriptional regulation, with BP terms such as specification of adaxial/abaxial axis, auxin-mediated signaling of phyllotactic patterning, maintenance of shoot apical meristem identity, post-embryonic morphogenesis and MF terms such as DNA-binding transcription factor activity showing enrichment, along with nucleus as the only enriched CC term (Supplementary Figure S2; Supplementary Table S7). For the late-responding genes, the MF terms protein-binding and catalytic activity were enriched, and enriched BP terms included response to diverse endogenous and exogenous stimuli including hormones and signaling, including hormone-mediated signaling. Enriched CC terms were the plasma membrane and cell periphery (Supplementary Figure S3; Supplementary Table S8). It is clear from these analyses that early-responding genes containing a putative STM-binding site were the most directly-related to shoot apical meristem development, primarily functioning at the level of transcriptional regulation, while the late-responding genes were involved in a more diverse array of functions connected with downstream processes such as cellular responses to hormones and other stimuli.

Given the enriched functions identified in the GO analysis, we next examined our ChIP and transcriptomics datasets to identify genes with known roles in meristem or lateral organ development, particularly those involved in the control of cellular differentiation and pluripotency or the regulation of plant hormone function. We found numerous genes with established roles in SAM development that were both bound by STM and showed an early transcriptional response to STM, with most of these showing sustained differential expression throughout the later stages of the time-course (Supplementary Table S5). Many of these encode transcription factors and include the meristem-associated *KNOX* genes *KNAT1/BP* and *KNAT2*, organ boundary-associated genes *CUP-SHAPED COTYLEDON1* (*CUC1*) and *LIGHT-SENSITIVE HYPOCOTYLS1* (*LSH1*), *LSH3*, *LSH4* and *LSH5*, and the *AP2*-related genes *AIL7/PLT7* and *AIL6/PLT3* and *RAP2.6L*. TF encoding genes associated with lateral organ development included the MYB family gene *ASYMMETRIC LEAVES1* (*AS1*) and the class-2 CIN-TCP gene *TCP4*, together with *BLADE-ON-PETIOLE1* and *BLADE-ON-PETIOLE2* (*BOP1* and *BOP2*). Other TF encoding genes associated with shoot development included the homeobox genes *HB2*, *HB25* and *HB32*, *MYB17*/*LMI2*, the *ASYMMETRIC LEAVES2*-related gene *ASL9*, and the growth regulating factor gene *GRF7*. Furthermore, we identified two genes involved in chromatin modification: *SUVR1*, which encodes a histone lysine methyltransferase, and *CHR40/CLASSY4*, which encodes an SNF2-domain protein that is involved in tissue-specific DNA methylation. In addition to transcriptional regulators, we also identified several genes encoding enzymes involved in auxin and cytokinin metabolism. These included the *MES9* and *MES10* genes, which encode methyl indole-3-acetic acid esterases, and the adenine phosphoribosyl transferase *APT2* and the cytokinin riboside 5’-monophosphate phosphoribohydrolase *LONELY GUY8* (*LOG8*).

A number of genes bound by STM showed a transcriptional response to STM only at the later timepoints in the time-course (Supplementary Table S6), and many of these were associated with plant hormone function, such as the auxin response factors *ARF4*, *ARF6* and *ARF10*, the auxin conjugating enzyme *GH3.3*, the *PINOID* kinase and the carboxylesterase *CXE6*, which has potential methyl indole-3 acetic acid esterase activity. In addition, two other adenine phosphoribosyl transferase enzymes, *APT3* and *APT4*, were detected in the late-responding dataset, together with the cytokinin response factor *CRF2* and the gibberellin associated genes *GA20OX3*, *GASA1* and *GID1B*.

Previous studies have also linked *KNOX* gene function, particularly that of *KNAT1/BP*, to cell wall metabolism^68–71^, especially secondary cell wall formation and lignification. In agreement with this, we identified several cell wall-associated genes in the late-responding dataset, including cellulose biosynthesis genes *CSLA11*, *CESA5*, *CSLG2*, xyloglucan metabolism genes *XTH24* and *XTH31* and pectin methylesterases *PME1*,* PME3* and *PMEPCRF*. The cellulose synthase *CSL11A* and pectin methylesterase *PMEPCRF* were also identified as early-responding target genes.

Finally, in the late-responding dataset we also identified the cell cycle regulator *CDKB2;1*, which has a role in shoot meristem development^72^, and the meristem-associated transcription factors *HB1*, *ATH1* and *BELL-LIKE HOMEODOMAIN4/ SAWTOOTH2* (*BLH4/ SAW2*), the latter two of which encode homeodomain proteins that physically interact with KNOX proteins including STM^73^.

Overall these results indicate that STM directly regulates a number of important factors in the control of development and hormone function. Many other genes potentially involved in meristem development, organ development or hormone function were identified in the ChIP-seq analysis yet did not show a transcriptional response to STM according to our transcriptomics analysis, or for which no transcriptional response data were available.

### STM gene regulatory network construction using bayesian network inference

Our analysis has revealed that genes encoding transcriptional factors (TFs) are significantly enriched among the STM-bound and STM-responsive target genes, suggesting that STM functions largely through controlling the expression of other transcriptional regulators. To explore the potential regulatory interactions among the STM-regulated transcriptional regulators and reveal the topology of the STM GRN, we performed Bayesian network analysis^74,75^ using the early responding STM-bound genes that encode transcriptional regulators as network components (nodes) and discretised data from ~ 2000 publicly available gene expression datasets to infer conditional dependency relationships to generate network edges. Though all these genes are likely to be directly regulated by STM, this approach allowed us to infer potential novel regulatory relationships and interplay between the various target genes and capture the potential functional sequence of these factors in regulating meristem function, as determined by the degree of separation (i.e. the number of intermediate nodes) from STM in the network. A total of 33 TF-encoding genes were selected for network construction (including STM; Supplementary Table S9), and the transcriptional response to STM was confirmed by qRT-PCR for a subset of these genes (Supplementary Figure S4), with most showing differential expression at short- and/or long-term timepoints following STM induction. Bayesian analysis was performed (50 iterations) to predict the frequency of conditional dependency relationships, indicated as edges between nodes (in both directions), and different frequency thresholds were explored to generate the consensus network, selecting > 30% confidence as the optimum threshold (i.e. conditional dependency relationships between two nodes being predicted in 30–100% of the 50 network iterations). Note that some genes formed mutual dependence relationships, which suggests feedback regulation. In these cases, the combined dependence frequencies for both directions should be considered.

**Fig. 5:**
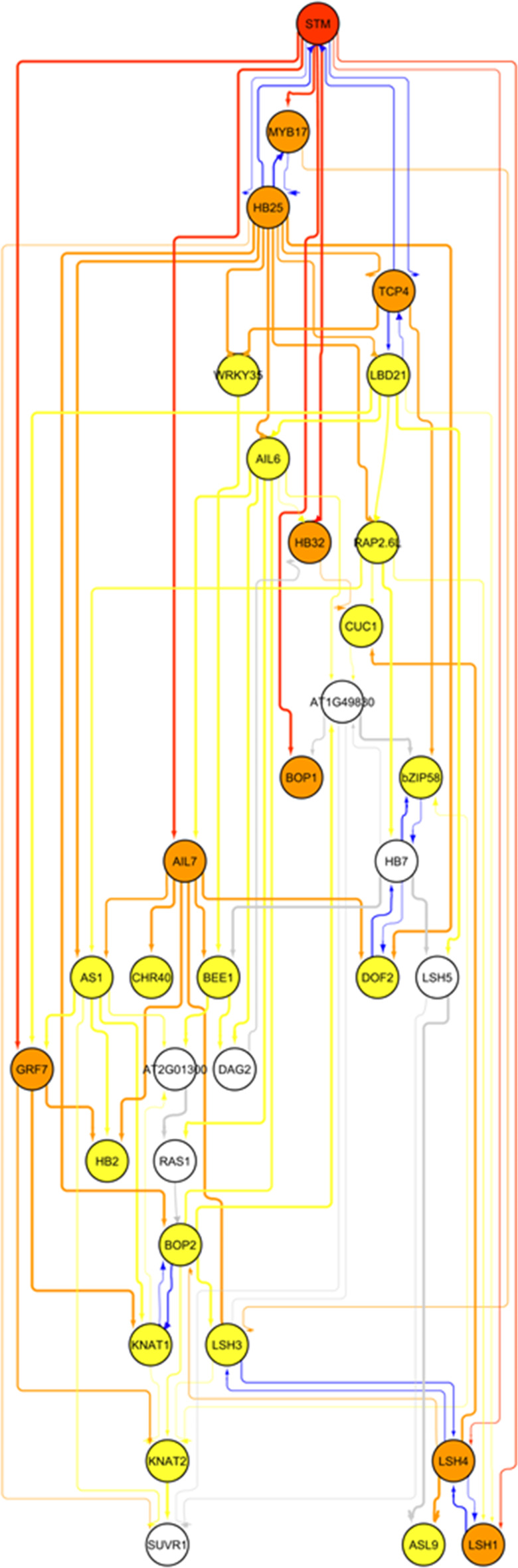
Bayesian network of STM target genes encoding transcriptional regulators. Nodes represent genes, and edges represent conditional dependency relationships. STM is shown in red, nodes directly connected to STM (1st order nodes) are shown in orange, nodes connected to these (2nd order nodes) are shown in yellow, and nodes connected to these (3rd order nodes) are shown in white. Edge connections use the same colour scheme. Edge thickness represents the connection threshold value (thinnest = 30% and thickest = 100%). Feedback relationship edges are highlighted in blue. Arrowheads on the edges indicate parent-to-child directionality.

The consensus network (Fig. 5) was able to capture several known regulatory relationships among the component genes, in the form of parent-child relationships, giving confidence to this approach. For example, *STM* and *TCP4* show mutual dependence, in agreement with the mutual transcriptional repression between these two genes^15,29^. Furthermore, *AS1* was shown to act as a parent to *KNAT1/BP*, which recapitulates the known transcriptional repression of *KNAT1/BP* by *AS1*^8–10^. Several members of the *LSH* gene family were connected to *CUC1* and *STM*, in agreement with their known role in comprising a meristem-organ boundary regulatory module^76^, together with *BOP2* which is also associated with regulation of *KNAT1/BP* and other *KNOX* genes^46,48^.

Our analysis reveals several genes that act as highly connected nodes, or hubs, within the network. As expected, *STM* itself is one of the most highly connected nodes, and shows direct connection to the boundary genes *LSH1*, *LSH4* and *LSH5*, which share potential feedback regulation with one another and themselves connect to CUC1, and several other genes involved in pluripotency, shoot formation and organogenesis (*AIL7*, *MYB17*, *BOP1*, *GRF7*, *HB32*, *HB25* and *TCP4*). *HB25*, a gene known to function in the promotion of shoot regeneration and development^50^, connects directly to several genes involved in multiple aspects of meristem function, such as pluripotency (*STM*, *AIL6*, *MYB17*, *RAP2.6L*), boundary formation (*CUC1* and *BOP2*) and organogenesis (*TCP4* and *AS1*). Moreover, *HB25* displays potential feedback relationships with *STM* and *MYB17*. Another key regulatory hub is *LBD21*, a member of the LOB-domain gene family which has no characterised function to date. *LBD21* connects directly to, and displays potential feedback with *TCP4*, and is also connected to *HB25*, *LSH1* and *LSH5*, *AIL6*, *RAP2.6L* and *GRF7*, which itself connects *STM* to *KNAT1/BP* and *KNAT2*. However, *LBD21* only showed a moderate transcriptional response to STM, so its importance must be treated with caution. Furthermore, the *AIL7* gene, which promotes pluripotency and controls phyllotactic patterning^39–41^, is also a highly-connected node in the network, with direct connections to *AS1*, the chromatin remodeller *CHR40* and the boundary gene *LSH3*, among others. Based on network analysis, we have also identified three genes (*HB7*, *HB32* and *At1g49830*, which encodes a bHLH TF) which may have important, hitherto unknown functions in the *LSH-CUC-BOP* meristem-organ boundary regulatory module. *HB32* connects *STM* to *CUC1*, and both *HB7* and *At1g49830* connect to *CUC1* and/or members of *LSH* gene family, suggesting possible involvement in this regulatory sub-network.

Overall, our Bayesian network analysis suggests a number of potential regulatory relationships between the various STM target transcriptional regulators and indicates that some may have more central roles than others in coordinating meristem function, based on their level of connectivity.

## Discussion

The class-1 *KNOX* genes encode TALE homeodomain proteins that are important regulators of shoot apical meristem development and function. A key function of the KNOX protein STM is to promote pluripotency and represses cellular differentiation in the cells of the SAM. Several studies have identified putative KNOX target genes in a variety of plant species, including rice, maize and Arabidopsis^25,26,29^. These studies have revealed that most KNOX target genes are associated with the control of hormone biosynthesis and response, cell differentiation and meristem organisation. In particular, STM has been shown to regulate a number of transcriptional regulators associated with meristem function^29,42^. Here, we expand this analysis to identify putative STM binding sites in the Arabidopsis genome using ChIP-seq analysis and correlate these sites with genes previously identified as transcriptionally responsive to altered STM levels. We also investigated the topology of the STM GRN using Bayesian network analysis. These combined approaches enabled us to develop and integrated picture of the regulatory interactions that take place among STM-regulated loci and allowed us to identify new genes involved in meristem function.

We utilised a robust approach for the identification of putative STM binding sites by comparing the STM-GR immunoprecipitated sample with both STM-GR no-antibody control and WT immunoprecipitated samples, which served as a control lacking the GR antigen. This approach was taken to eliminate noise and reduce the number of false-positive peak predictions, and substantially reduced the number of putative binding sites identified compared to the use of only a no antibody control. Hence, our analysis is conservative and there may be additional STM binding sites in the Arabidopsis genome which were excluded using this approach.

Our ChIP-seq analysis revealed 858 peaks corresponding to 859 distinct loci which contain one or more putative binding sites for STM. These binding sites were mostly located around the TSS or < 1 kb upstream of the TSS, though some binding sites were located further upstream and others were detected in, or downstream of, the gene body. This is in broad agreement with previous studies^25^. Assigning a locus to a particular peak was done on the basis of the closest gene to the peak, but was not always straightforward, as some peaks were detected in intergenic regions that were approximately equidistant from two adjacent loci. In such cases, we assigned the peak to whichever of the two genes showed a transcriptional response to increased STM levels using our previously published transcriptomics data^29^. If both were responsive or unresponsive to STM, then the peak was assigned to both loci (6 genes). Hence, we made the assumption that peaks were associated with the transcriptional control of the closest locus, but this does not take into account potential long-range effects. Moreover, identification of transcriptionally responsive genes was done using a rather limited dataset generated using Affymetrix ATH1 microarrays, which does not contain probe-sets for all transcripts from the Arabidopsis genome. Therefore, comparison of binding sites with global RNA-seq datasets should be performed to gain a more comprehensive identification of STM-responsive genes. Though many genes were identified by ChIP-seq that did not show a transcriptional response to STM, or for which no data were available, we confine the following discussion primarily to those genes which were both bound by STM and showed differential expression in response to altered STM activity, as these are the most likely to represent functionally important STM targets.

Identification of putative KNOX target genes has led to the discovery of conserved *cis*-regulatory elements in target gene promoters. Canonical homeodomain proteins such as Antennapedia in *Drosophila melanogaster* typically bind to *cis*-regulatory elements containing a TAAT core in target gene promoters or enhancer elements^59^. Likewise in plants, the WOX homeodomain protein WUS also has affinity for TAAT sequences but has also been shown to bind the sequence TGAA and the G-box sequence TCACGTGA^60–64^. TALE homeodomain proteins preferentially bind to *cis*-regulatory elements containing a different core motif, such as the core TTAC motif bound by the yeast protein Matα2^77^. Previous studies have shown that class-1 KNOX proteins in plants have high affinity for *cis*-acting sequences that contain one or more TGAC cores. This was first shown for the HVH21 KNOX protein in barley^56^, and subsequently for NTH15 in tobacco^33^ and KN1 in maize^57^. Further studies revealed that many KNOX-bound *cis*-regulatory elements comprised two TGAC cores, separated by a small number of nucleotides. For example, KN1 binds to a motif composed of two TGAC cores in the first intron of the *GA20ox1* gene^55^ while in potato a similar motif was bound by POTH1 in the promoter region of *Ga20ox1*^34^. In rice, OSH1 bound a motif containing two TGAC cores in its own promoter and in that of other rice *KNOX* genes^58^, while in Arabidopsis, STM was shown to bind a motif comprising two TGAC cores in the promoter of the *CUC1* gene^42^.

We searched for conserved motifs within the sequences comprising peaks in the STM-ChIP experiment using a hidden Markov model-based approach. We were able to identify 5 distinct motifs within STM ChIP peaks, each containing one or more TGAC or TGAT cores. Of these, 3 were dyads comprising TGAC + TGAC, TGAT + TGAT or TGAT + TGAC motifs, while two contained a single TGAC core. Motif 4 was the longest motif, with several A or G residues present immediately after the TGAC core. The presence of motifs containing a TGAT core may indicate that KNOX proteins, or KNOX-BEL heterodimers, also have binding affinity for this sequence. Our motif prediction may have been too stringent to capture all biologically relevant *cis*-regulatory sequences, as we were unable to detect any of these motifs in the upstream promoter of *CUC1*, despite the presence of multiple TGAC sequences with the STM-bound region. However, the strong overrepresentation of TGAC motifs within the STM-bound sequences suggests that, as in other species, this motif forms the core of most STM-bound *cis*-regulatory elements STM target gene promoters or other regulatory regions. Future studies utilising in vitro binding assays, such as electrophoretic mobility shift assays, could help to understand the STM binding affinities for each of these *cis*-regulatory motifs.

Given that KNOX proteins have affinity for such a generic and widespread binding motif, it has been proposed that binding partners are involved in conferring target gene specificity. Indeed, KNOX proteins form heterodimeric complexes with members of the BLH (BEL1-like homeodomain) family of TALE homeodomain proteins, comprising 13 members in Arabidopsis^78–80^. Specificity in the regulation of target genes could therefore rely on the composition of these heterodimeric complexes, which also facilitate nuclear import, with different KNOX and BELL partners interacting to recognise different *cis*-regulatory elements using nucleotides outside of the TGAC core^34^. Furthermore, WUS has been shown to heterodimerise with STM and bind to the regulatory sequences of stem cell-signaling gene *CLV3*, with such STM binding also captured in our ChIP-seq data, highlighting the importance of such cooperative regulatory interactions^61,81^.

From a functional perspective, the STM-bound target genes identified here were in broad agreement with those found in maize and rice^25,26^. We found that many of the target genes which showed a transcriptional response to STM at early timepoints in our transcriptomics data are known regulators of SAM function. In particular, our data showed direct STM regulation of other *KNOX* genes (*KNAT1/BP* and *KNAT2*), in agreement with previous studies, but did not detect STM itself as an STM-bound target, whereas the rice ortholog OSH1 promotes its own expression as well as that of other *KNOX* genes^58^. Our network analysis also captured AS1-mediated regulation of *KNAT1*^8–10^, and revealed GRF7 as a potential novel factor involved in the regulation of *KNAT1*,* KNAT2* and the homeobox gene *HB2*.

Several genes involved in the formation of meristem-organ boundaries were identified, including *CUC1*^42^, which also activates *STM* expression during embryogenesis^43–45^, together with several members of the *LSH* gene family whose expression is also activated by *CUC1*^76^. These data support the known role for STM in organ boundary specification and previously established transcriptional regulatory relationships^29,82^. Our Bayesian network analysis identified direct connections and feedback relationships between several members of the *LSH* gene family, *STM* and *CUC1*. Though STM directly activates *CUC1* expression, the network only predicted a direct connection between these two factors at 20% confidence, which might result from the fact that *CUC1* expression is activated independently of STM during embryogenesis. Instead, *CUC1* showed that connection to *STM* is routed through *LSH4* and *HB32*, the latter of which may represent a novel component of the boundary regulatory module. Indeed, two additional genes (*At1g49830*, encoding a bHLH transcription factor, and the homeobox gene *HB7*) were identified that showed network connections to *CUC1* and/or the *LSH* genes and may represent additional new components of this regulatory module.

STM was also shown to directly regulate genes involved in pluripotency, shoot regeneration and phyllotaxis. These included three members of the AP2-related family of transcription factors. These were *RAP2.6L*, which has an established role in shoot development and regeneration^83,84^, and the *AINTEGUMENTA-like/PLETHORA* genes *AIL7/PLT7* and *AIL6/PLT3*, which are involved in promoting pluripotency in the SAM and in controlling phyllotactic patterning^39–41^. Hence, their functions overlap with those of *STM* suggesting they may be important downstream mediators of STM function in SAM development. In support of this, *AIL7*, and to a lesser extent *AIL6* and *RAP2.6L*, were highly connected hub nodes within the network, and showed connections to numerous other genes with functions including boundary formation, *KNOX* gene regulation, hormone function and chromatin regulation.

Several genes involved in lateral organ formation were also identified, and many of these the functioned in both the repression of *KNOX* gene expression and the promotion of differentiation and organ polarity. *BLADE-ON-PETIOLE1 (BOP1)* and *BOP2* were directly bound by STM and were transcriptionally induced in response to increased STM activity. BOP1 and BOP2 function to promote leaf adaxial identity and have been shown previously to repress the expression of *KNAT1/BP*, a relationship that is also captured in our Bayesian network analysis, together with *KNAT2* and *KNAT6*, while activating expression of *AS2*, the *LATERAL ORGAN BOUNDARIES* (*LOB*) gene and *ATH1*, which encodes a BELL domain protein that interacts with STM^46–49,85^. Furthermore, we have previously shown that STM downregulates several members of the class-2 *TCP* gene family^29^, which function in the repression of *KNOX* gene expression and the promotion of differentiation in leaf primordia. Here we show that STM binds upstream of the *TCP4* promoter, though not the promoters of the related genes *TCP3* and *TCP10*, suggesting only regulation of *TCP4* is direct. Indirect down-regulation could be mediated by promoting expression of repressors of CIN-TCP expression, such as the *miR319* gene family, for example^86^. Together with the known repression of *KNOX* gene expression by TCPs^15^, this provides a mutually antagonistic mechanism by which the *KNOX* and *TCP* expression domains can be delineated, ensuring robust separation of meristem and organ primordium identities during development. In support of this, our network analysis captured the potential mutual regulatory relationship between *STM* and *TCP4*.

Chromatin structure regulation has been shown to be important in the control of STM expression during floral development^87,88^. However, little is known about how STM might itself regulate chromatin at target loci. Here, we identify two chromatin regulatory factors as targets of STM: *SUVR1*, which encodes a histone lysine methyltransferase and *CHR40/CLASSY4*, which encodes an SNF2-domain protein that is involved in tissue-specific DNA methylation. *CHR40* expression has been shown to be localised to the SAM, suggesting that it has specific roles related to meristem function, and acts to control RNA-directed DNA methylation^89,90^. SUVR1 forms a complex with other chromatin remodelling proteins and is involved in transcriptional gene silencing through changes in nucleosome positioning^91^. Hence, these two factors may be involved in the control of STM target gene accessibility to the transcriptional machinery, both at the level of DNA methylation and nucleosome positioning.

STM has previously been implicated in the control of phytohormone biosynthesis, perception and signaling, especially relating to cytokinin, auxin and GA. STM promotes expression of the IPT7 gene^32,36^, which encodes an isopentyl transferase enzyme that is required for CK biosynthesis, in addition to the CK-receptor AHK4, and represses expression of cytokinin oxidase (CKX) genes^22^. We did not detect binding of STM to these loci, suggesting such regulation may be indirect. However, we did detect direct regulation of the cytokinin riboside 5’-monophosphate phosphoribohydrolase gene *LONELY GUY8* (*LOG8*), which is involved in converting cytokinin nucleotide conjugates to the active free-base form^92^, and adenine phosphoribosyl transferases *APT2-4*, which catalyse the reverse reaction^93^. We further show that STM also binds to and transcriptionally regulates several genes involved in the auxin pathway, with STM targets including several methyl indole-3-acetic acid esterases and carboxylesterases, which catalyse the conversion of methyl-IAA to the free IAA form. Additional auxin-associated factors included several genes encoding auxin response factors (ARFs), *SAUR71*, the auxin-conjugating enzyme GH3.3 and the kinase *PINOID*. For GA associated genes, we detected STM binding to genes encoding a GA20 oxidase, a GA2 oxidase and a GA3 oxidase, though only *Ga20ox3* showed a transcriptional response. Paradoxically, this gene showed up-regulation in response to STM in the later timepoints, suggesting that this may be some kind of feedback regulation or compensation mechanism to restore GA homeostasis arising from the severely altered morphology of these plants. In agreement with this, we detected binding and up-regulation of the GA-responsive gene *GAS1* and the GA-receptor *GID1B* at later timepoints following STM up-regulation. We also identified an additional STM target gene, *BRANCHED-CHAIN AMINO ACID TRANSAMINASE2* (*BCAT2*), which involved in regulation of both *KNOX* gene expression and the biosynthesis of GA and cytokinin^94,95^.

Overall our combined analysis of genes that are both bound by STM and show a transcriptional response to increased levels of STM activity, together with our Bayesian network analysis, has revealed many directly-regulated STM target genes that include known transcriptional regulators and hormone-associated genes that have critical roles in controlling pluripotency and cellular differentiation associated meristematic activity and lateral organ formation. We also identified several genes with hitherto unknown regulatory roles that may be biologically important in the control of meristem function that make promising targets for future functional studies.

## Materials and methods

### Plant lines and growth conditions

The transgenic p35S::STM-GR line was provided as a gift from Rüdiger Simon^20^. WT Columbia-0 (Col-0) seedlings were used as the no-GR epitope control for ChIP. Seeds were germinated on GM medium (4.4 g/L MS salts, 0.5% MES buffer, 1.5% sucrose, 1% microagar) supplemented with 60 µM dexamethasone (DEX) and grown under 24 h continuous white light at 22^o^C for 11 days. Shoot tissue was harvested for ChIP experiments, and root tissue was discarded. All plant research was conducted within the Plant Growth Technology Hub in the Cardiff School of Biosciences in compliance with international and UK guidelines. No endangered species were used in this research.

### Chromatin immunoprecipitation (ChIP) and DNA sequencing

ChIP was performed according to Morohashi et al. (2009)^96^ starting with 1 g of homogenised aerial tissue of 11 day-old p35S::STM-GR plants^20^ and WT Col-0 plants (no GR-epitope control) grown on GM agar containing 60 µM DEX. Chromatin fragmentation was performed using a Covaris M220 focused ultrasonicator. Protein A agarose beads (Merck) were used to pre-clear the digested chromatin followed by the anti-glucocorticoid receptor alpha antibody PA1-516 (Thermo Fisher) for immunoprecipitation (IP) or BSA for no-antibody mock-immunoprecipitation (NoAB) controls. Two biological replicates were performed for each sample type (as per ENCODE guidelines). DNA from IP and NoAB chromatin samples was recovered using a Qiagen MinElute DNA Purification Kit alongside an input sample consisting of fragmented chromatin that was not immunoprecipitated. DNA concentration was measured using a Qubit fluorometer. Paired-end DNA libraries were prepared with NEXTflex Rapid DNA-Sequencing Kit 1.0 (BioScientific) using the low input protocol and quality was checked using a D1000 ScreenTape (Agilent). Sequencing was performed on the Illumina Next Seq 500 System by the Biosciences Genomics Research Hub within the School of Biosciences, Cardiff University.

### ChIP-seq data analysis

Low quality reads, sequencing artefacts, and adaptors were removed using fastp v0.20^97^. Sample quality reports were produced using FastQC (v0.11.9) and summarised with MultiQC v1.9^98^. Trimmed FASTQ reads were mapped to the Arabidopsis thaliana TAIR10 reference genome^99^ using Bowtie 2 v2.4.1^100^with a maximum fragment length of 500 bp. ChIP signal distribution was visually assessed in IGB^101^ using BigWig files generated with deepTools v3.3.0^102^. Peak calling was performed with MACS2 v2.2.4^103^ for each IP and NoAB sample using their respective input sample as a background control. ChIPQC v1.26.0 within R v4.0.2^104^ was used to assess peak calling quality. DiffBind v3.17^105^ was used to identify statistically enriched peaks in each IP sample using their respective NoAB as a control and DESeq2 with an FDR < 0.05. BEDTools intersect v2.29.2^106^ was used to discard all peaks present in the no GR-epitope control. The resulting putative binding sites were annotated using ChIPseeker v1.27.3^107^ and the Araport11 gene annotation^108^.

### Gene set enrichment analysis (GSEA)

Gene set function was assessed by performing Gene Ontology term enrichment using g: Profiler g: GOSt^109^ with Ensembl version 109 and a g: SCS threshold of 0.05. GSEA plots were generated using custom R scripts within R version 4.3.2 (https://github.com/TamaraLechon/Visualisation/blob/main/go_lollipop_charts.R).

### Binding motif analysis

Motif enrichment analysis was performed within the RSAT Plants Suite^110^. Sequences for each of the peaks were retrieved from their respective genomic coordinates. RSAT peak-motifs was used to perform motif discovery^111,112^. Positional analysis (position-analysis and local-word-analysis) was performed with a hidden Markov model of order 2 and no background model, restricted to 6 and 7 bp oligomers and only 3 hits per algorithm. Word analysis (oligo-analysis and dyad-analysis) was performed with a Bernoulli model and a background model built of random Arabidopsis thaliana genome sequences, restricted to 100 bp around the peak summit, 6 and 7 bp oligomers and only 3 hits per algorithm. The resulting motif matrices were clustered to group similar motifs together using RSAT matrix-clustering^113^.

### Bayesian network structural inference

Gene regulatory network analysis was performed using static Bayesian machine learning from discretised microarray expression data as described in Scofield et al. (2018)^29^. 2372 microarrays annotated for seedling or shoot tissue were retrieved and discretised using a custom R script within R version 4.3.2 (https://github.com/TamaraLechon/Bayesian-network-inference/blob/main/microarray_discretisation.R). The discretised matrix was used as input data to infer a consensus gene regulatory network using Bayesian Network Inference with Java Objects (BANJO) v2.2.0 with the simulated annealing algorithm. Networks were run for 1 h, with a maximum parent count of 10, over a maximum of 10,000 restarts, initial simulated annealing temperature of 10,000, a cooling factor of 0.7, reannealing temperature of 800, a maximum of 2,500 accepted networks before cooling, a maximum of 10,000 proposed networks before cooling and a minimum of 500 accepted networks before reannealing. Fifty top-scoring networks were generated and a consensus network was produced via influence scores with a threshold of 30%. Networks were visualised using Cytoscape v3.7.2^114^.

### qRT-PCR analysis

Total RNA was isolated with Tripure (Roche) and cDNA synthesis was performed using the Ambion Retroscript kit or the Protoscript II kit (New England Biolabs). qRT-PCR was conducted on the Rotorgene 6000 RT-PCR machine (Qiagen) using qPCRBIO SyGreen Lo-Rox Mix (PCR Biosystems) and ACTIN2 as reference. Data was analysed using the ∆∆CT method^115^. 35:STM-GR^20,29^ or 35S:TGV; pTF:STM^29^ lines treated with 60µM DEX were used for short-term (3 h) or longer-term (from germination) experiments respectively. For short-term experiments, target gene expression in DEX treated samples was compared to mock (DMSO) treated samples. For longer-term experiments, a DEX-treated empty vector control line (designated 237) was compared to 35S:TGV; pTF:STM lines, with two independent 35S:TGV; pTF:STM lines (designated S34 and S38) used for analysis. Averages and standard deviations (error bars) from multiple experiments (2–6 independent biological replicates) are shown in Figure S4, together with data from individual replicates. Primer sequences are available in Supplementary Table S10.

## Electronic supplementary material

Below is the link to the electronic supplementary material.

Supplementary Material 1

Supplementary Material 2

Supplementary Material 3

Supplementary Material 4

Supplementary Material 5

Supplementary Material 6

Supplementary Material 7

Supplementary Material 8

Supplementary Material 9

Supplementary Material 10

Supplementary Material 11

Supplementary Material 12

Supplementary Material 13

Supplementary Material 14

Supplementary Material 15

Supplementary Material 16

## Data Availability

All sequencing data are deposited in the NCBI Sequence Read Archive under BioProject ID PRJNA1167216.
