# Supplementary material for: Regulation of meristem and hormone function revealed through analysis of directly-regulated SHOOT MERISTEMLESS target genes
Source: Sci Rep. 2025 Jan 2;15:240. doi: 10.1038/s41598-024-83985-1 (PMC11696002; doi:10.1038/s41598-024-83985-1)
Supplement: Supplementary file 11 — Supplementary Material 11 [file 41598_2024_83985_MOESM11_ESM.docx]

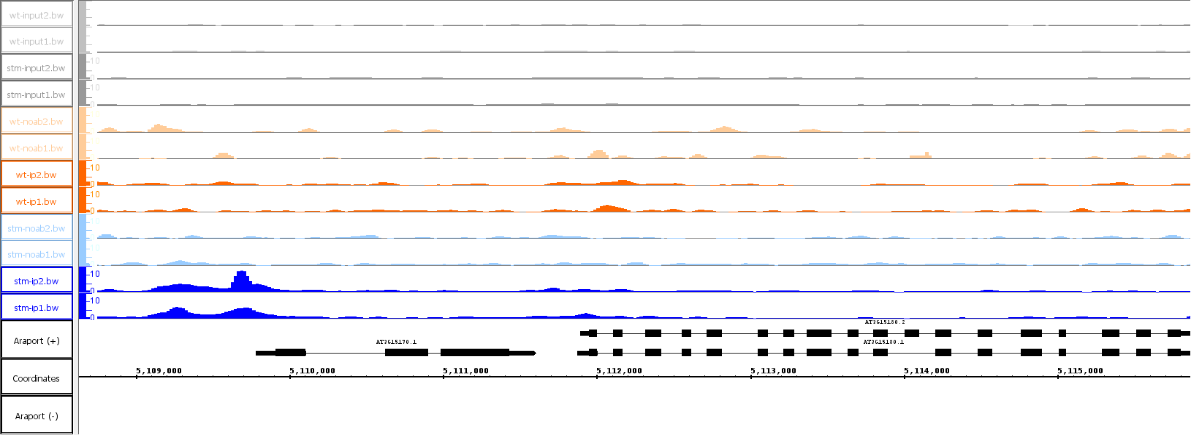


CUC1


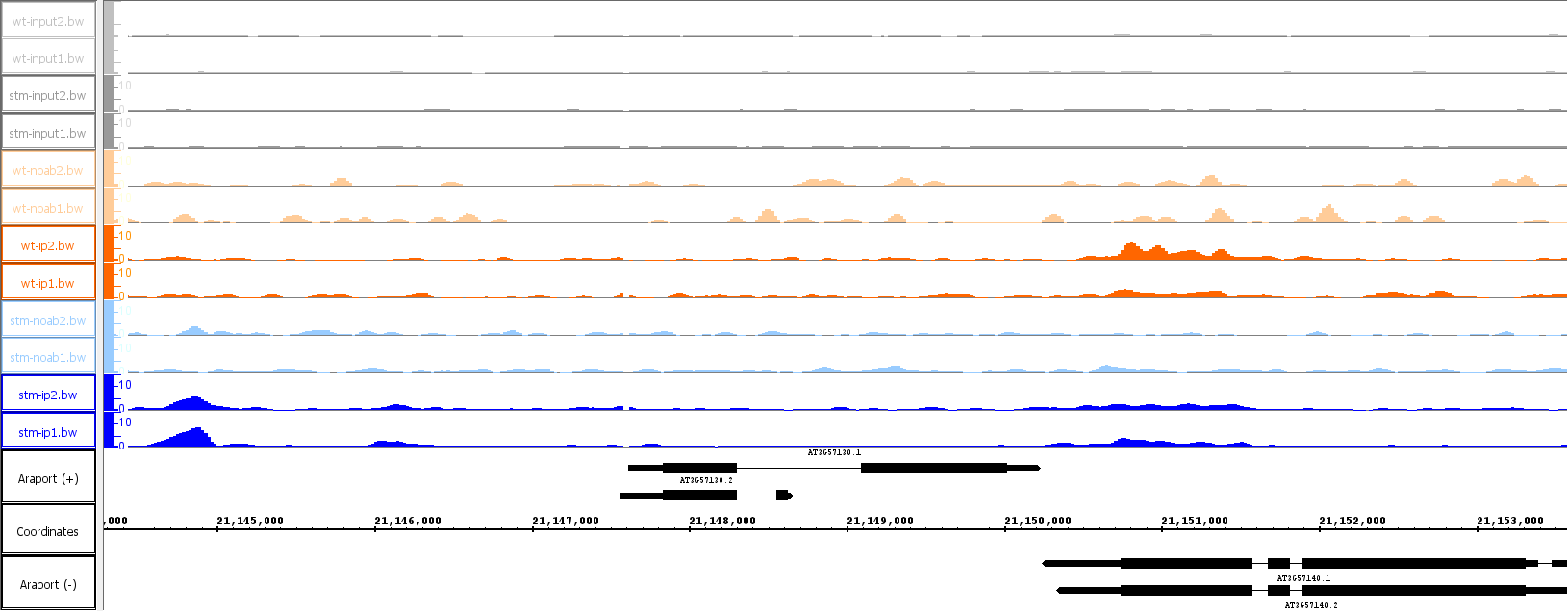


BOP1


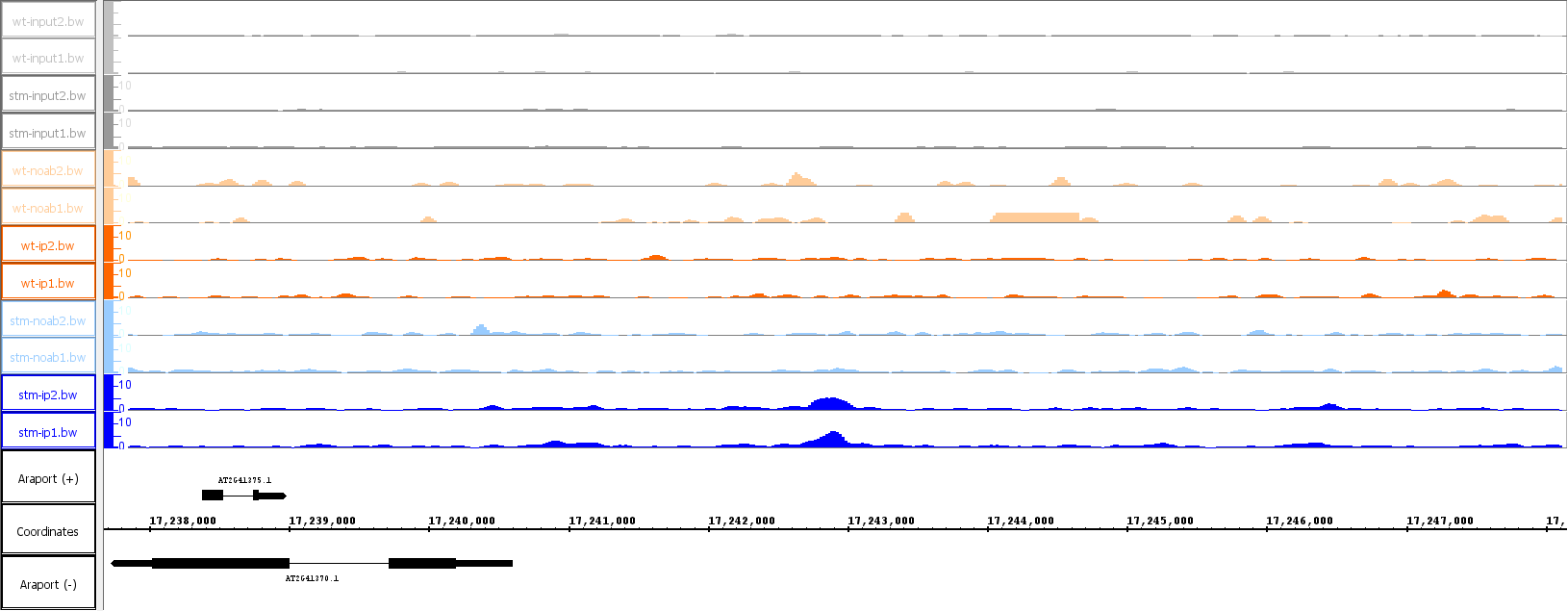


BOP2

CHR40


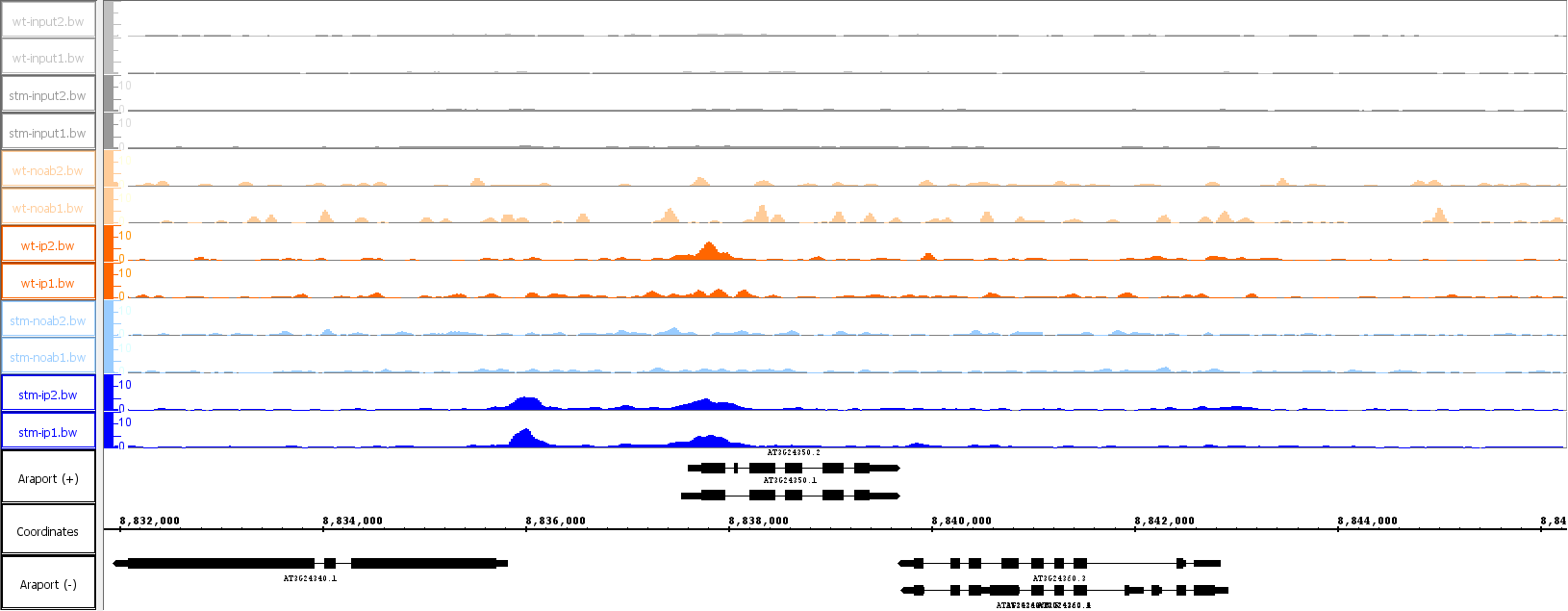


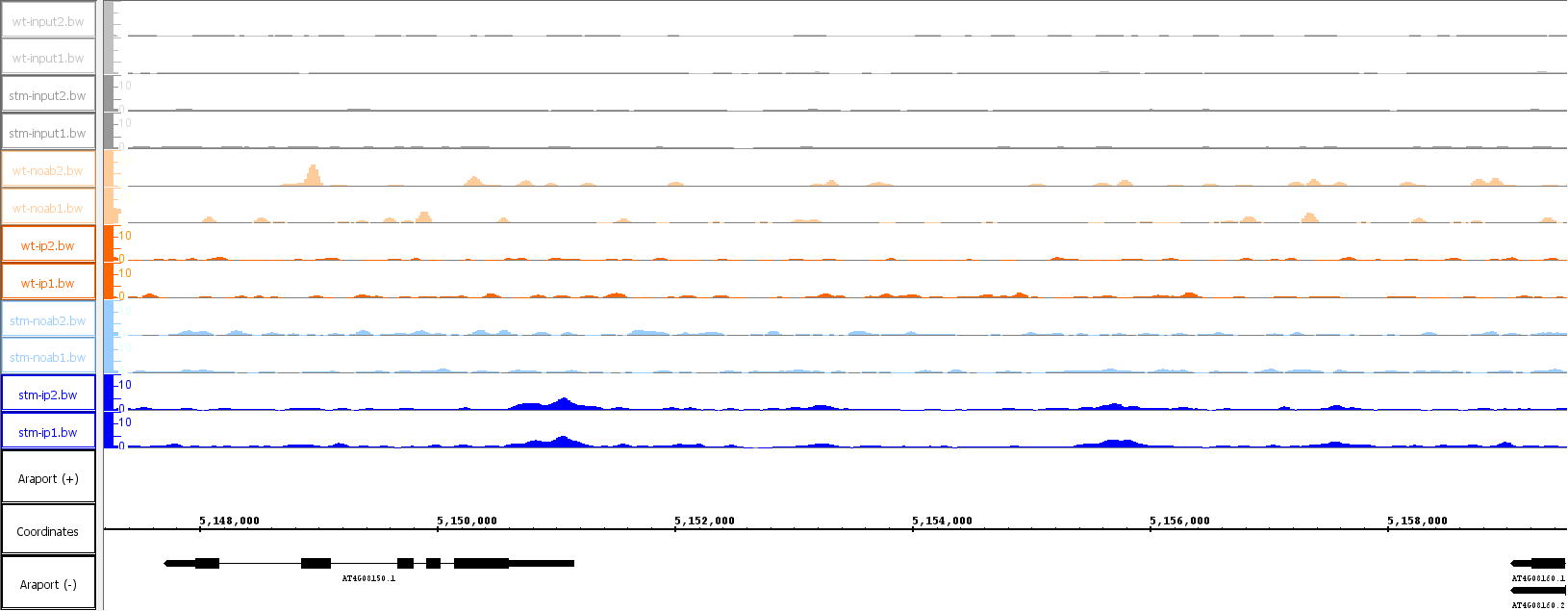


KNAT1


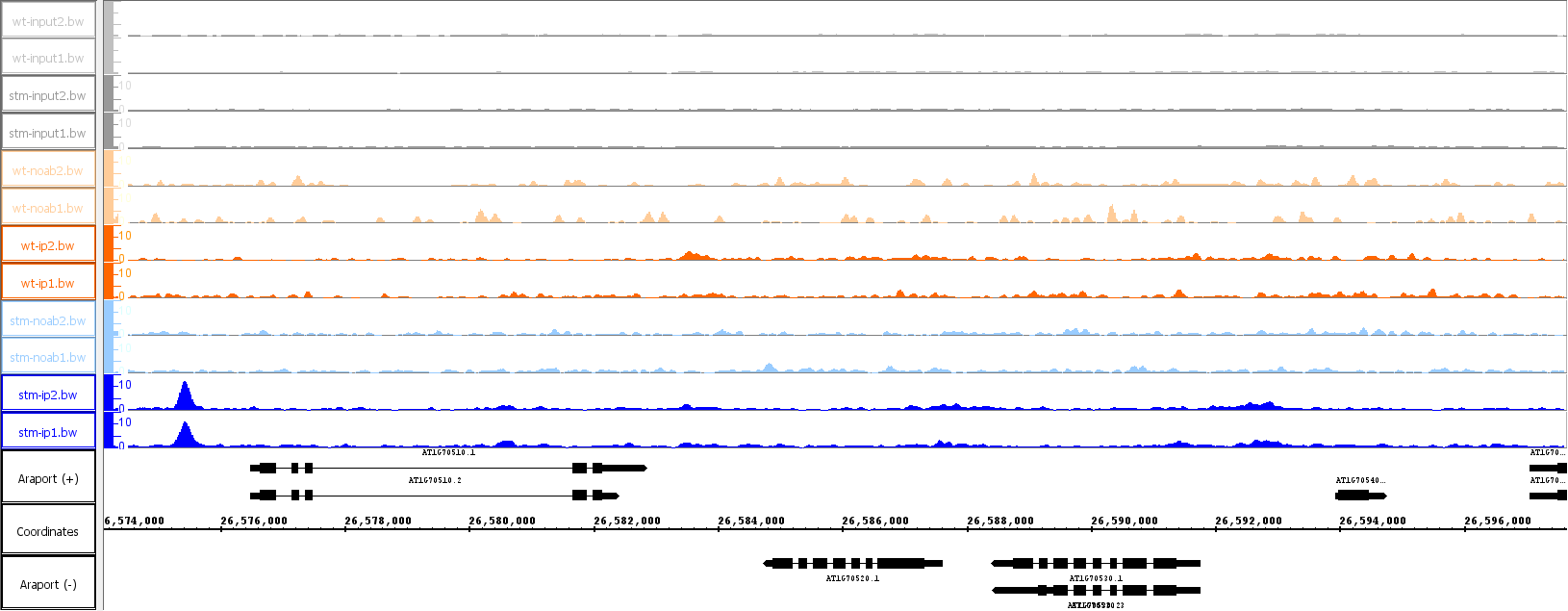


KNAT2


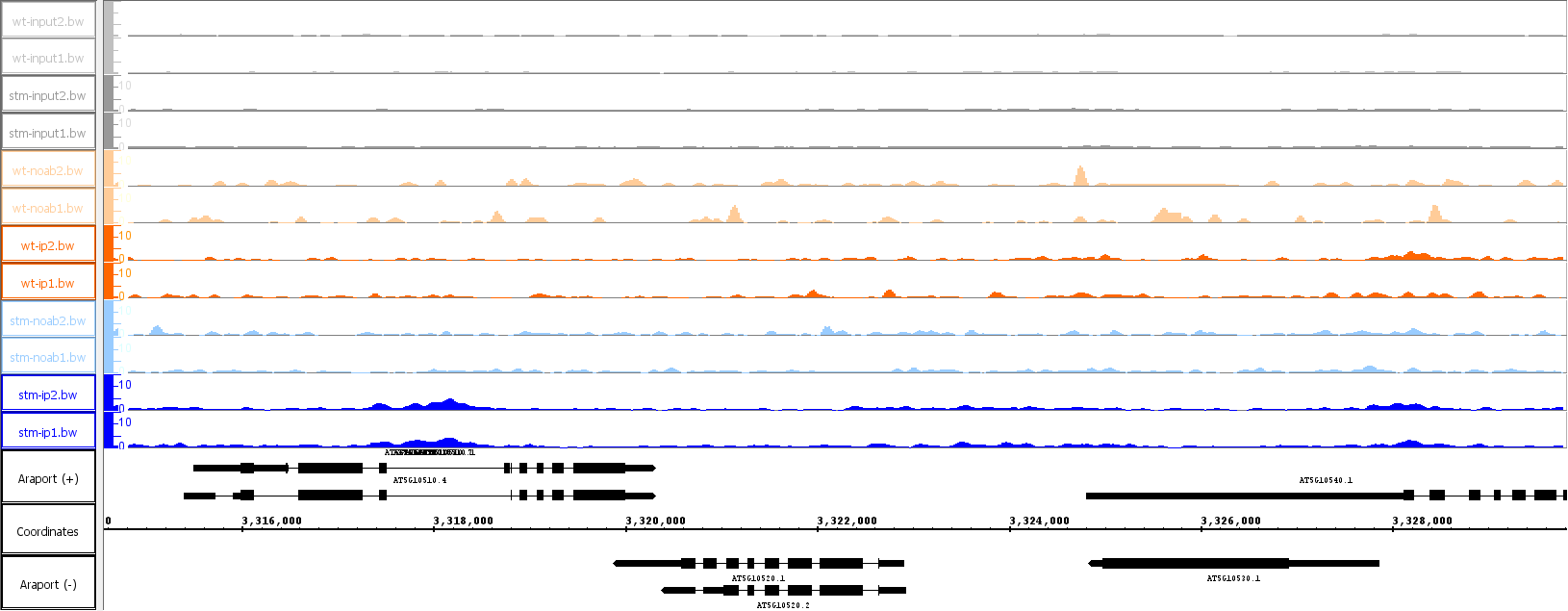


AIL6


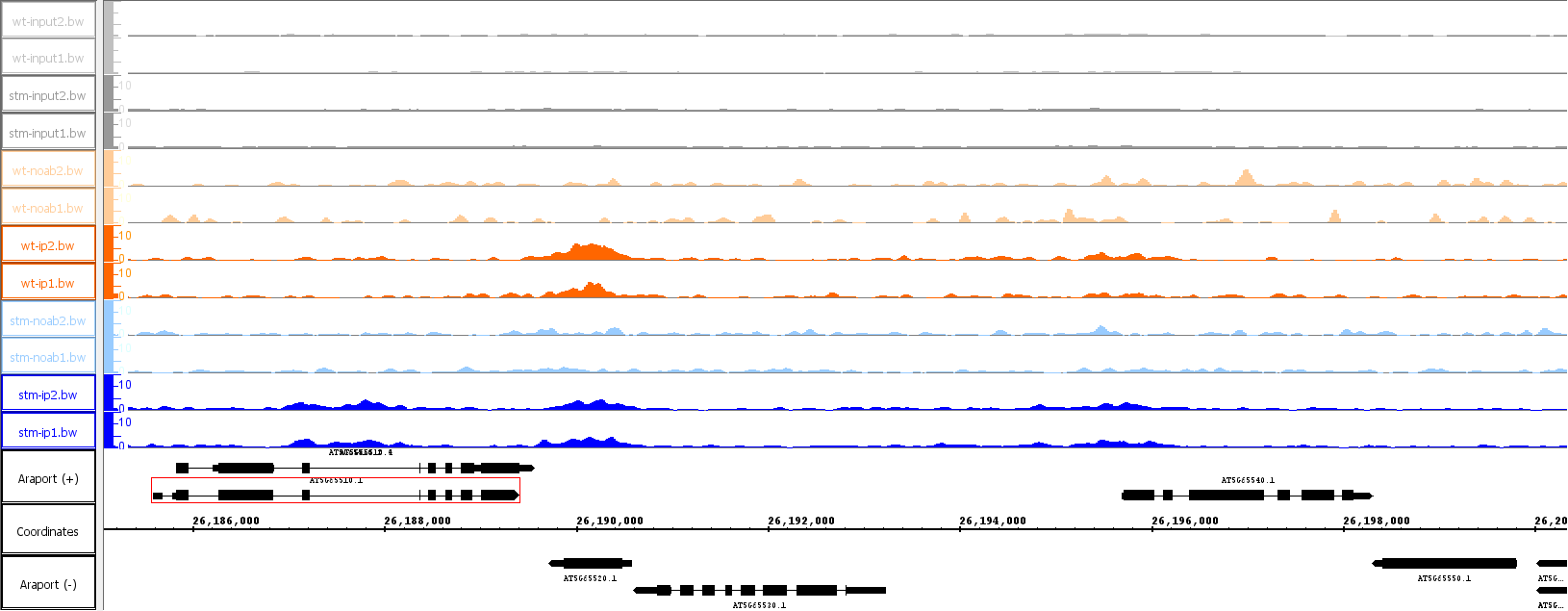


AIL7


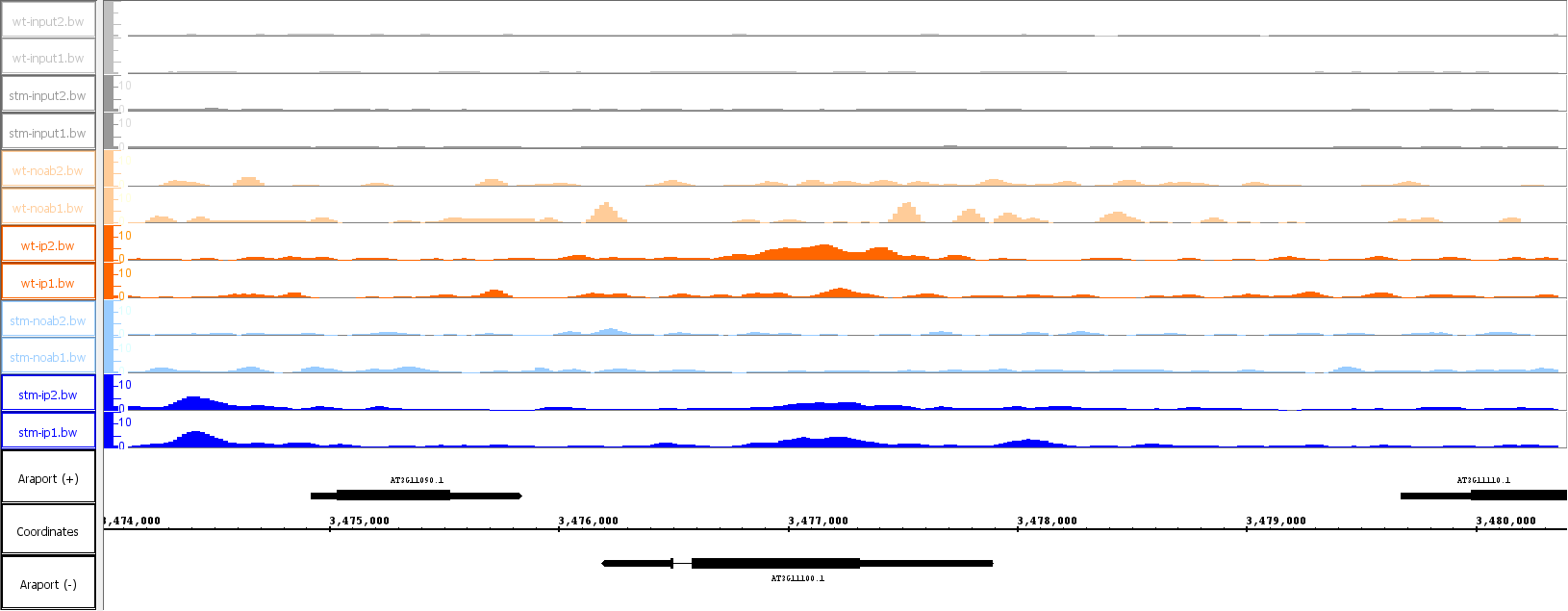


LBD21

HB25


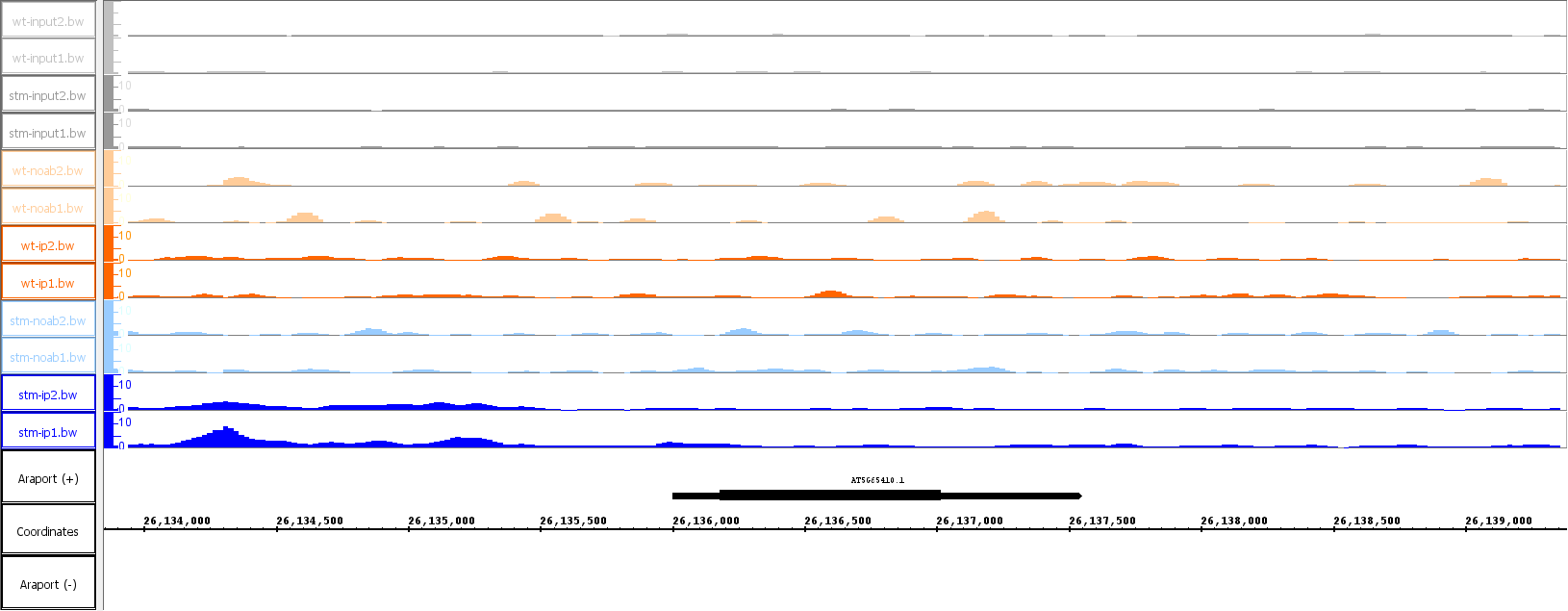


HB32


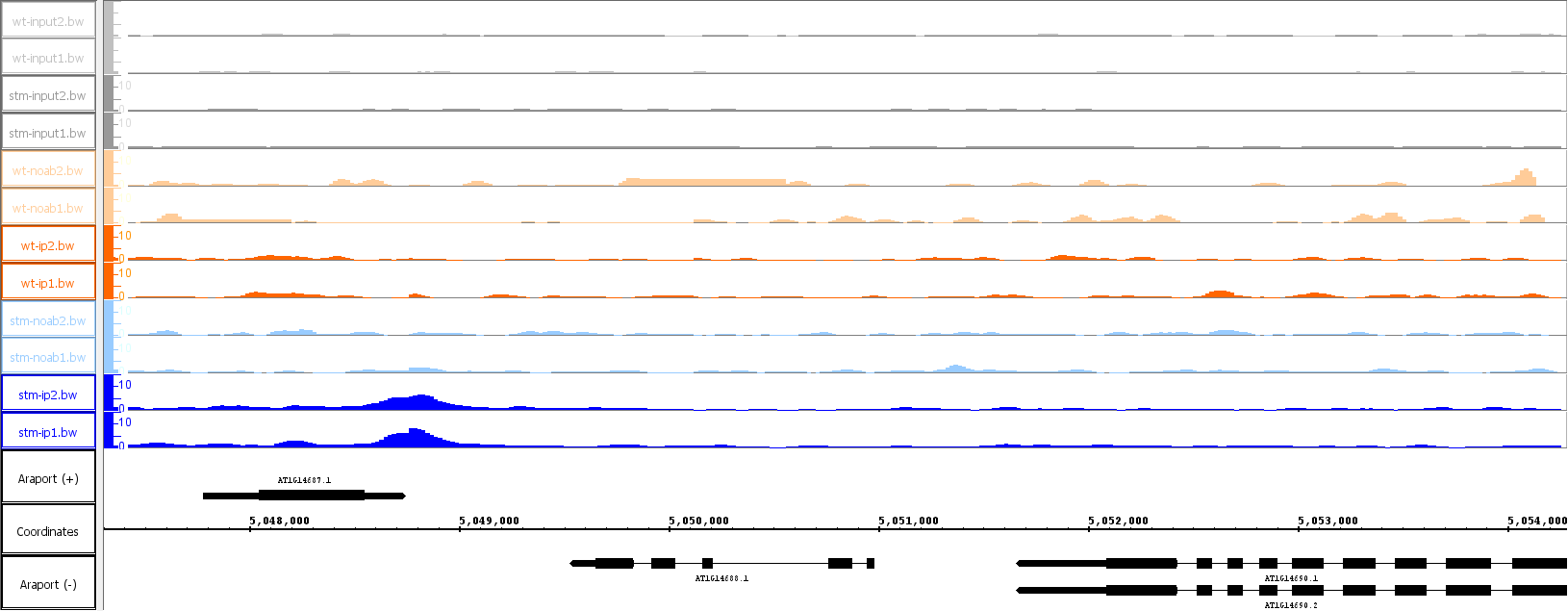


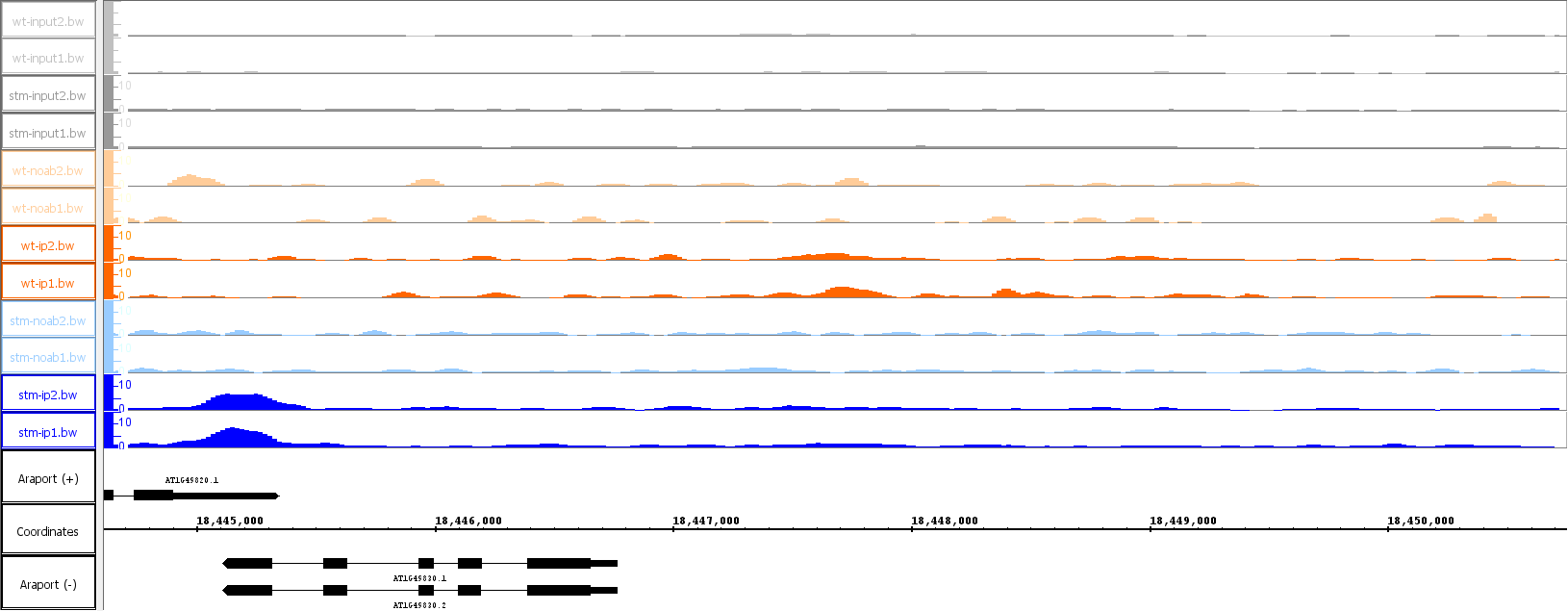


At1g49830


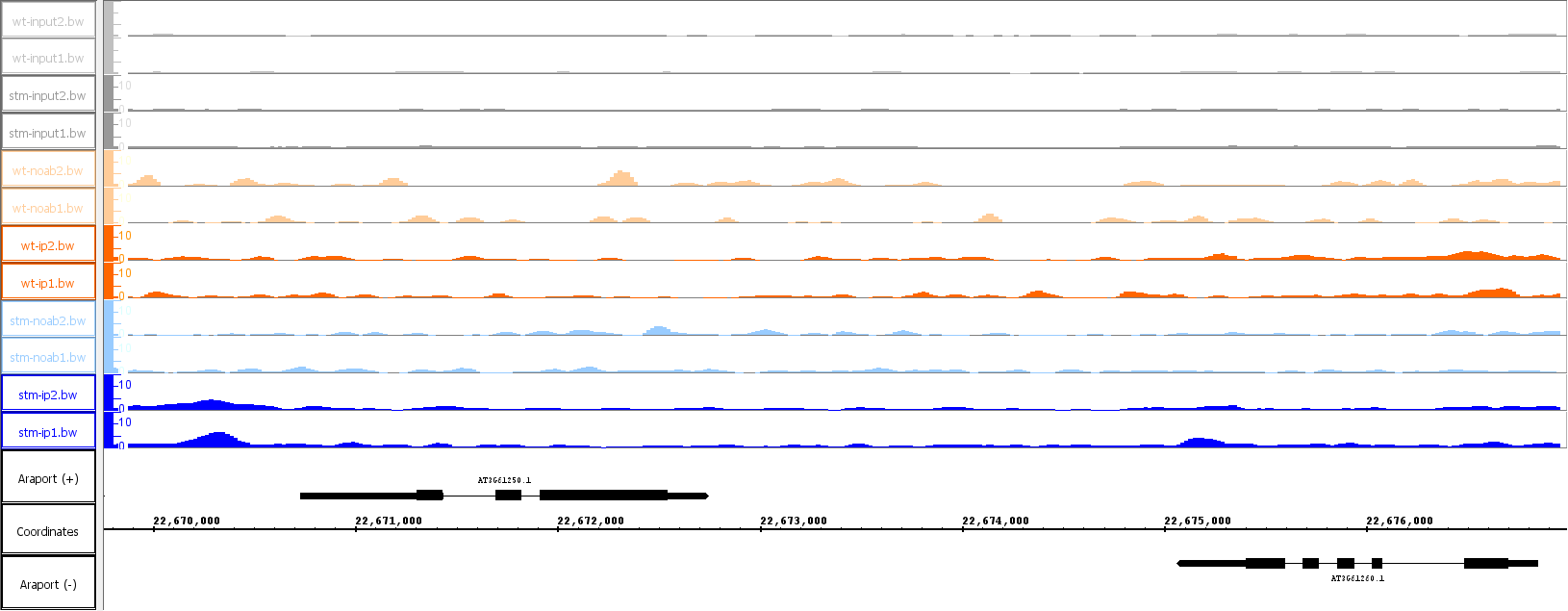


MYB17


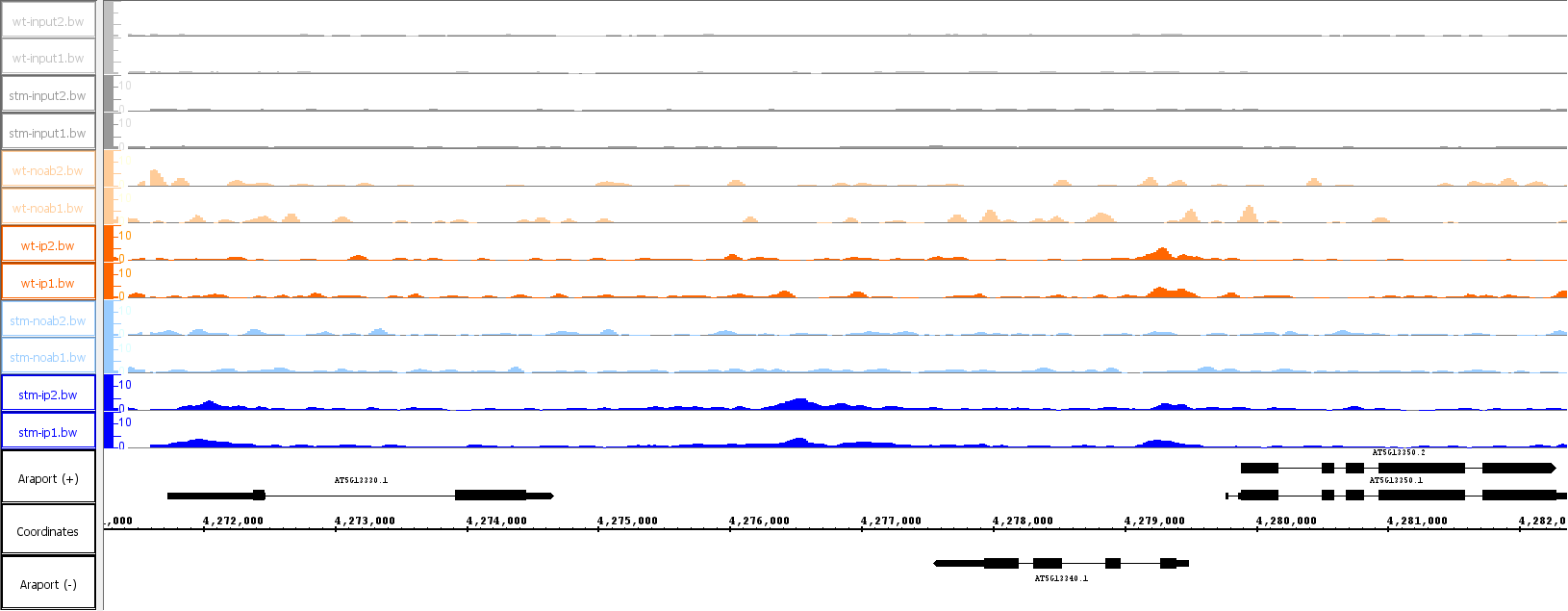


RAP2.6L

AS1


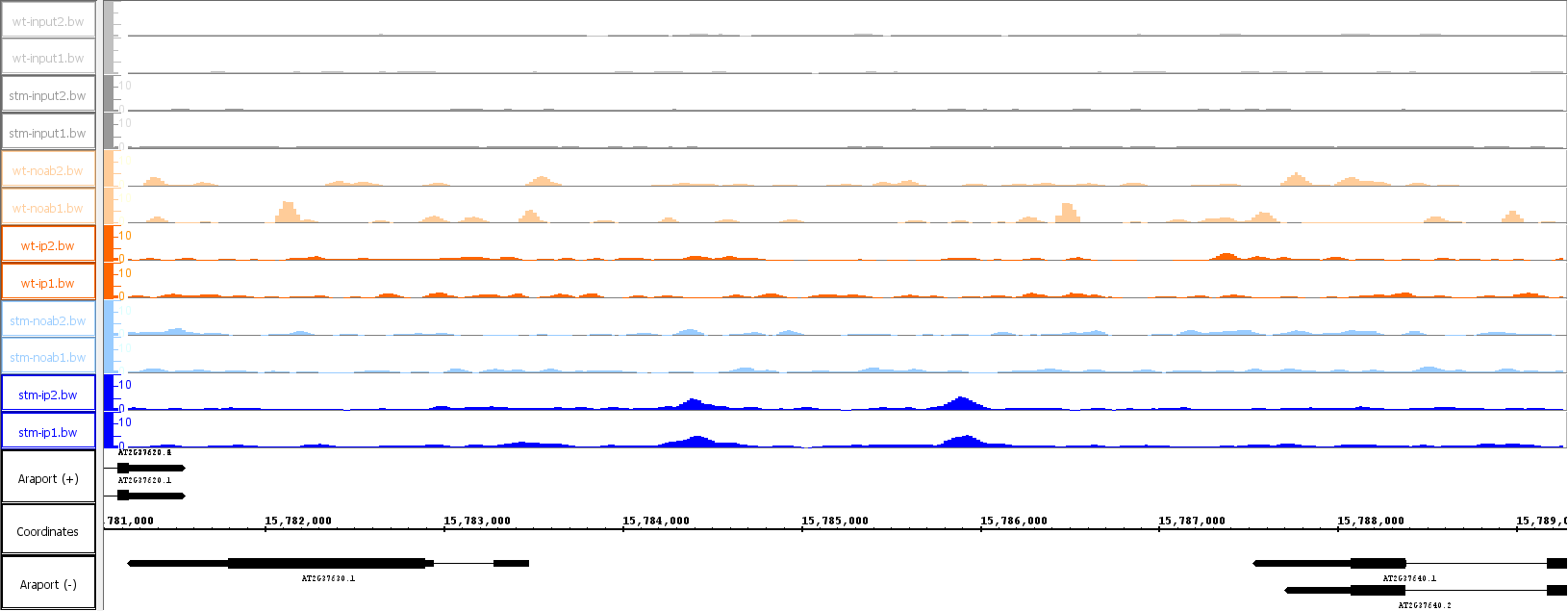


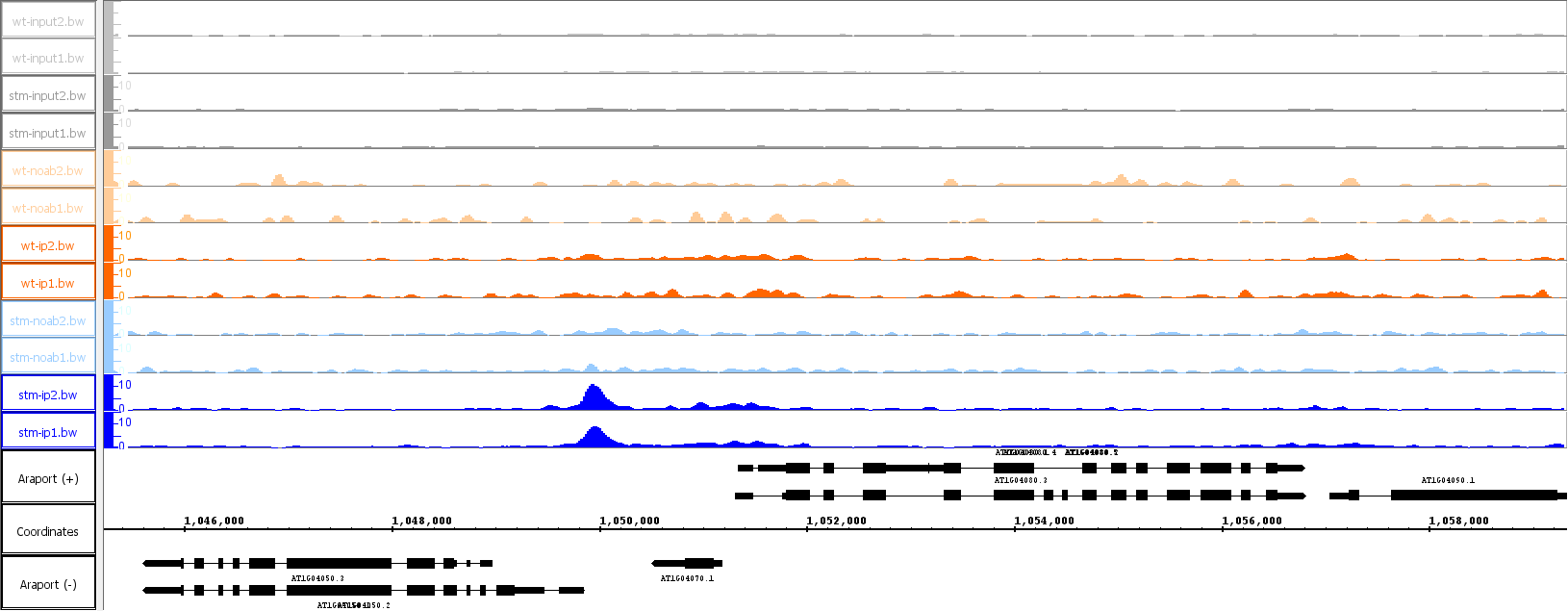


SUVR1


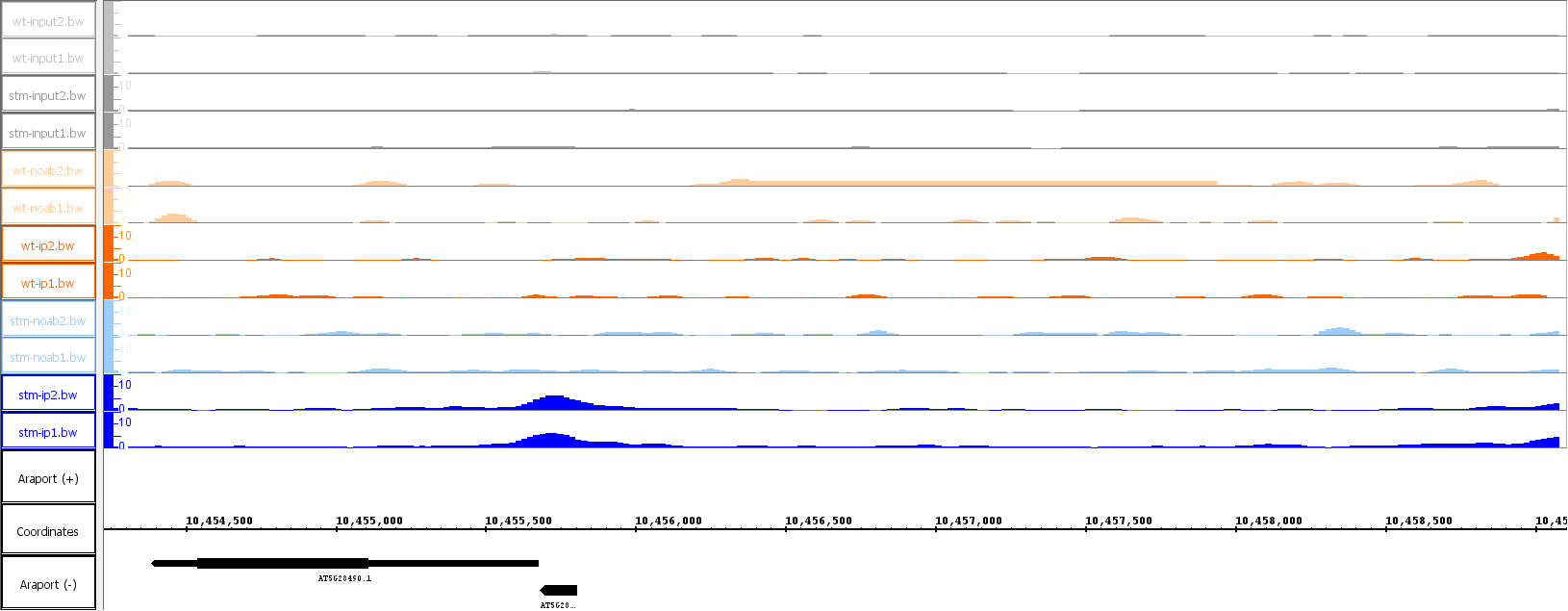


LSH1


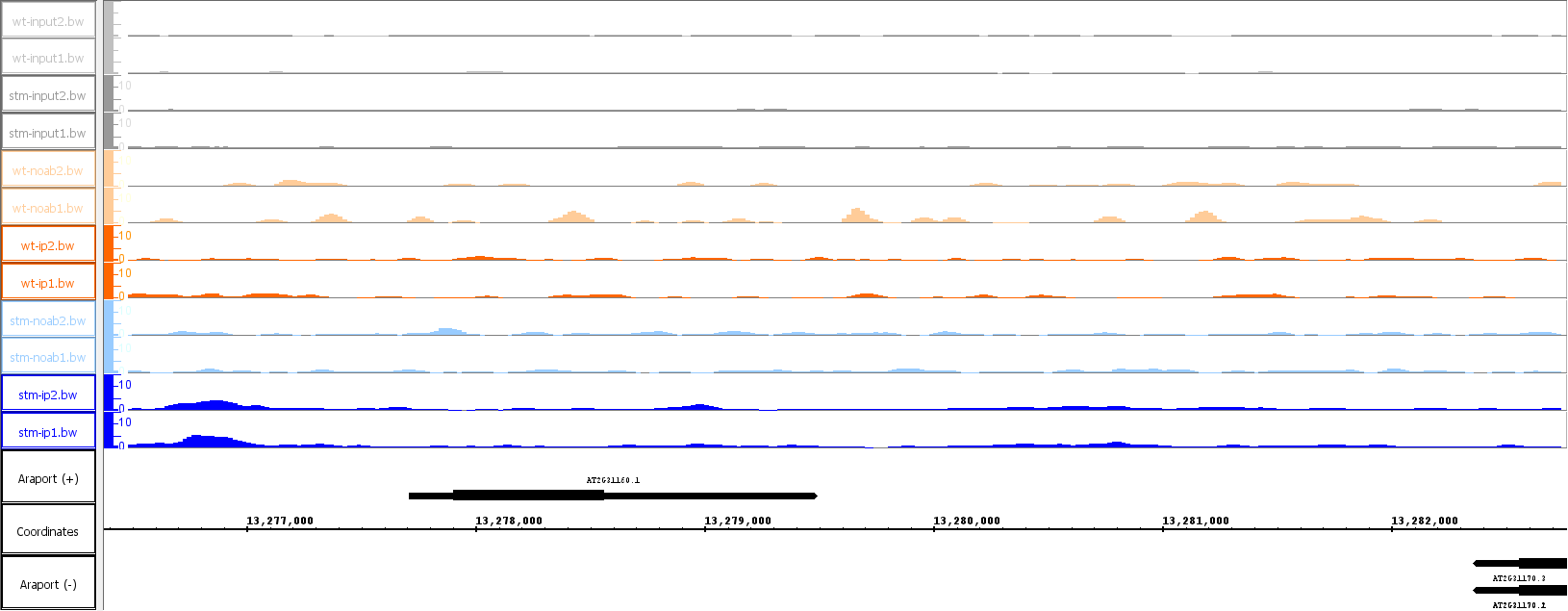


LSH3


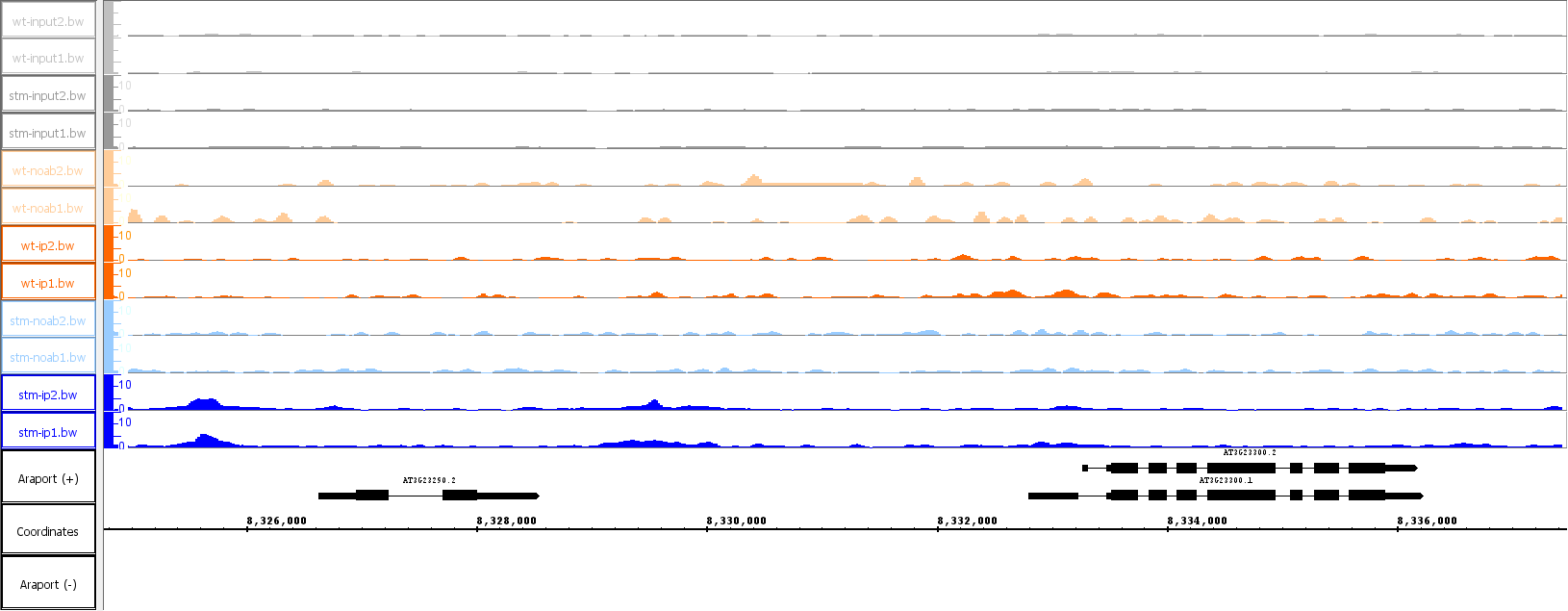


LSH4


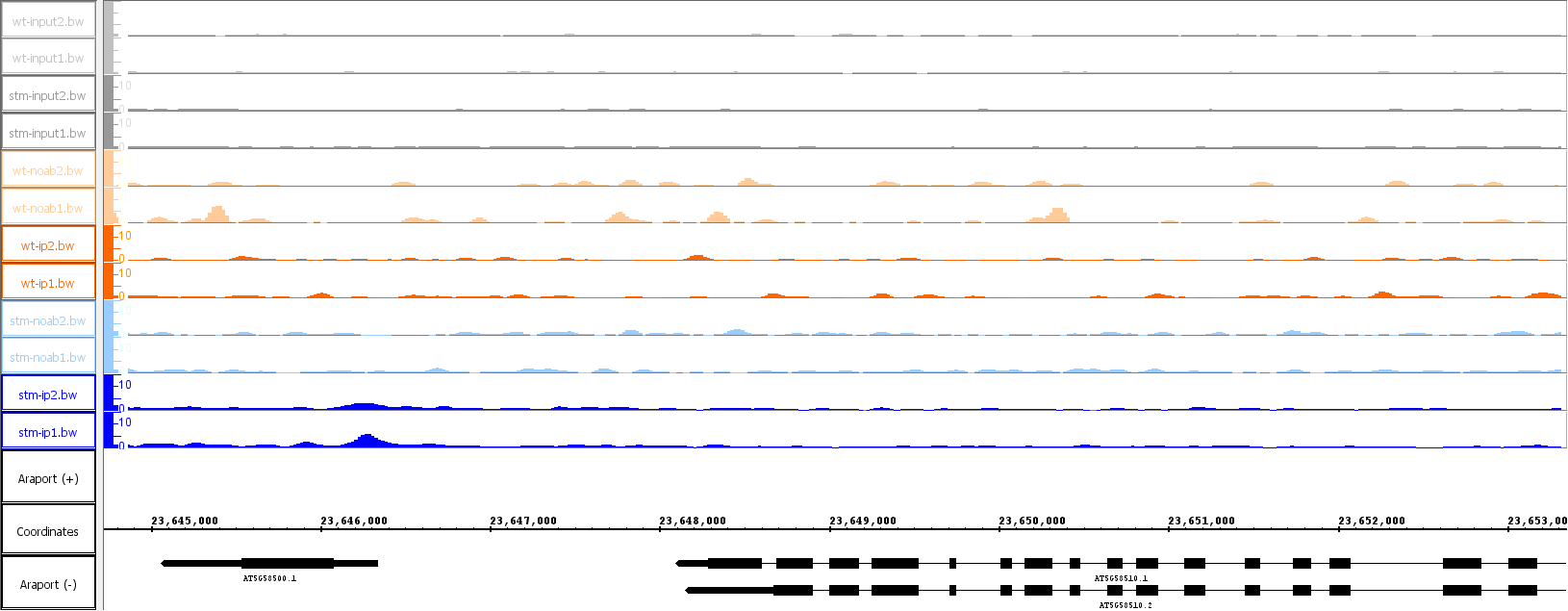


LSH5

TCP4


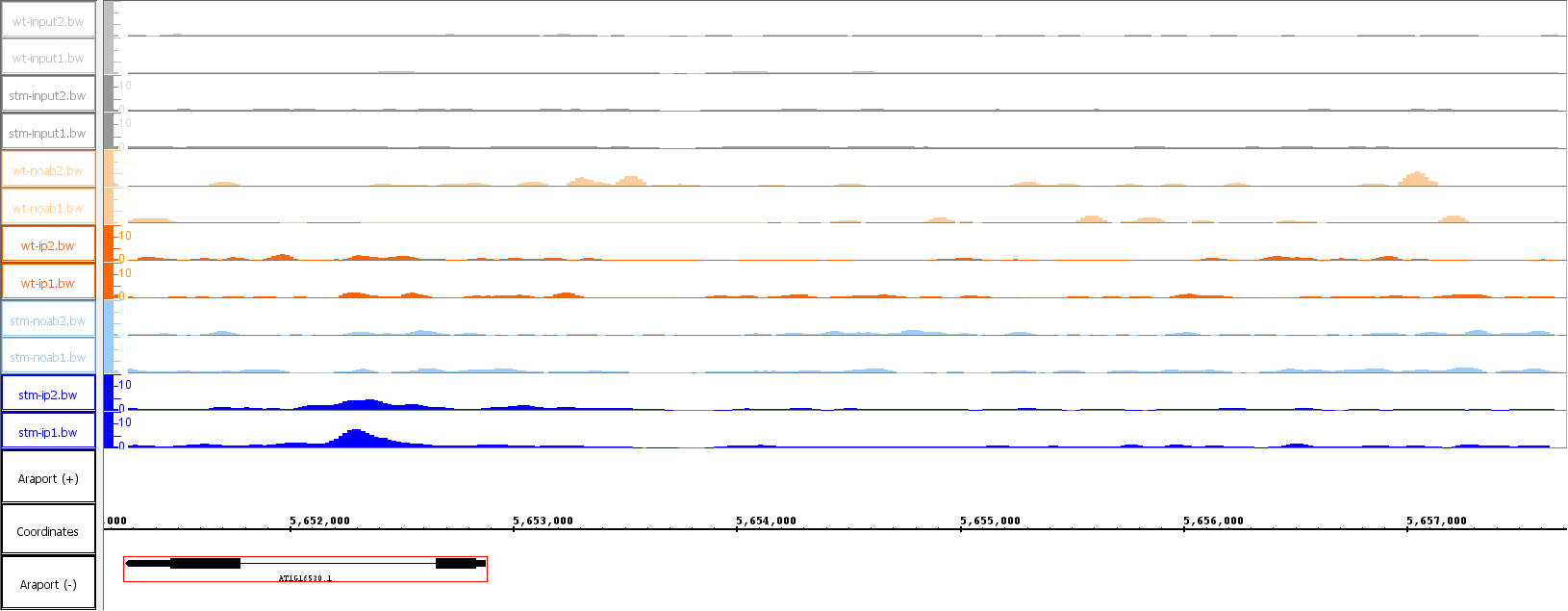


ASL9


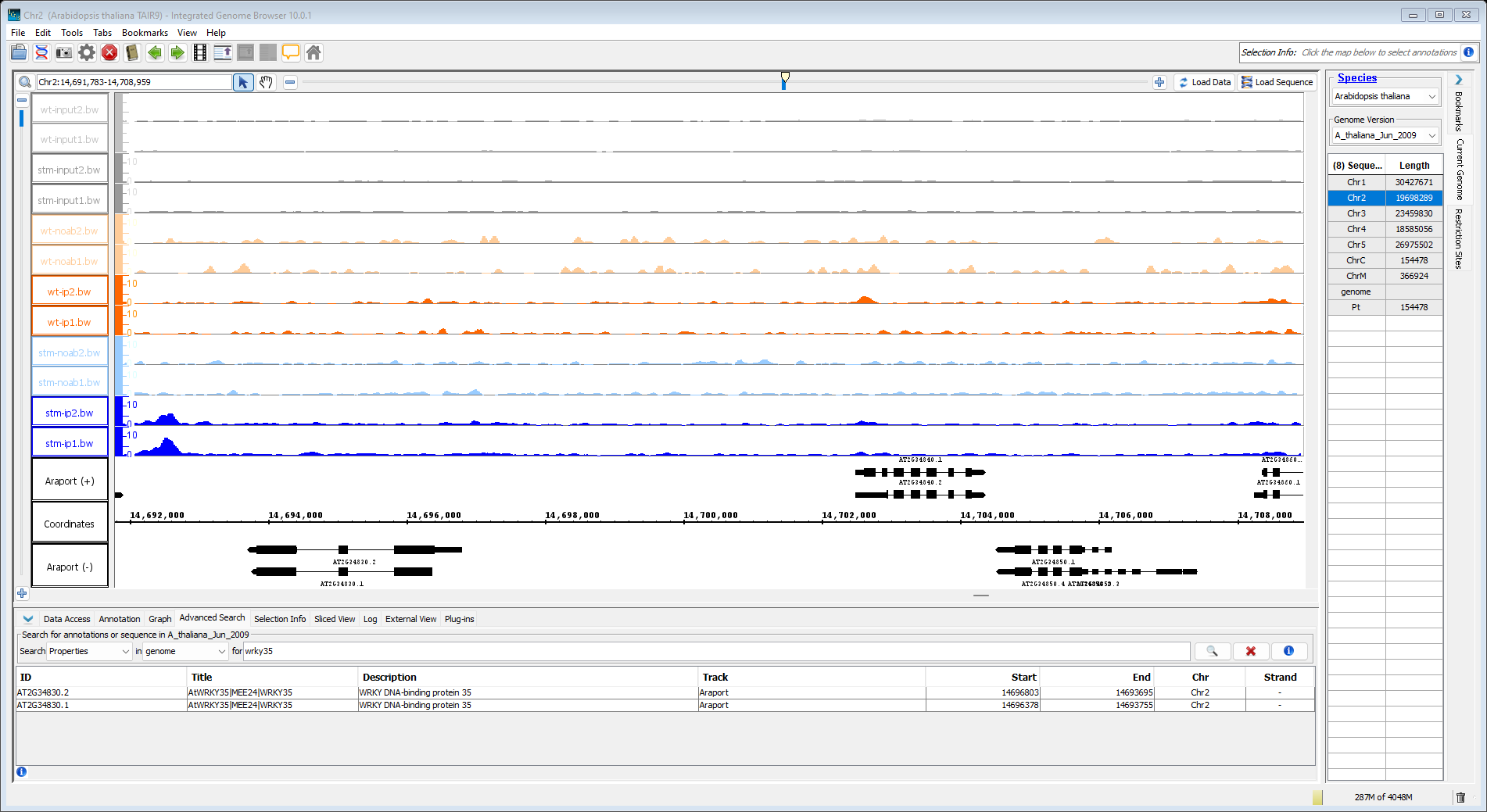


WRKY35


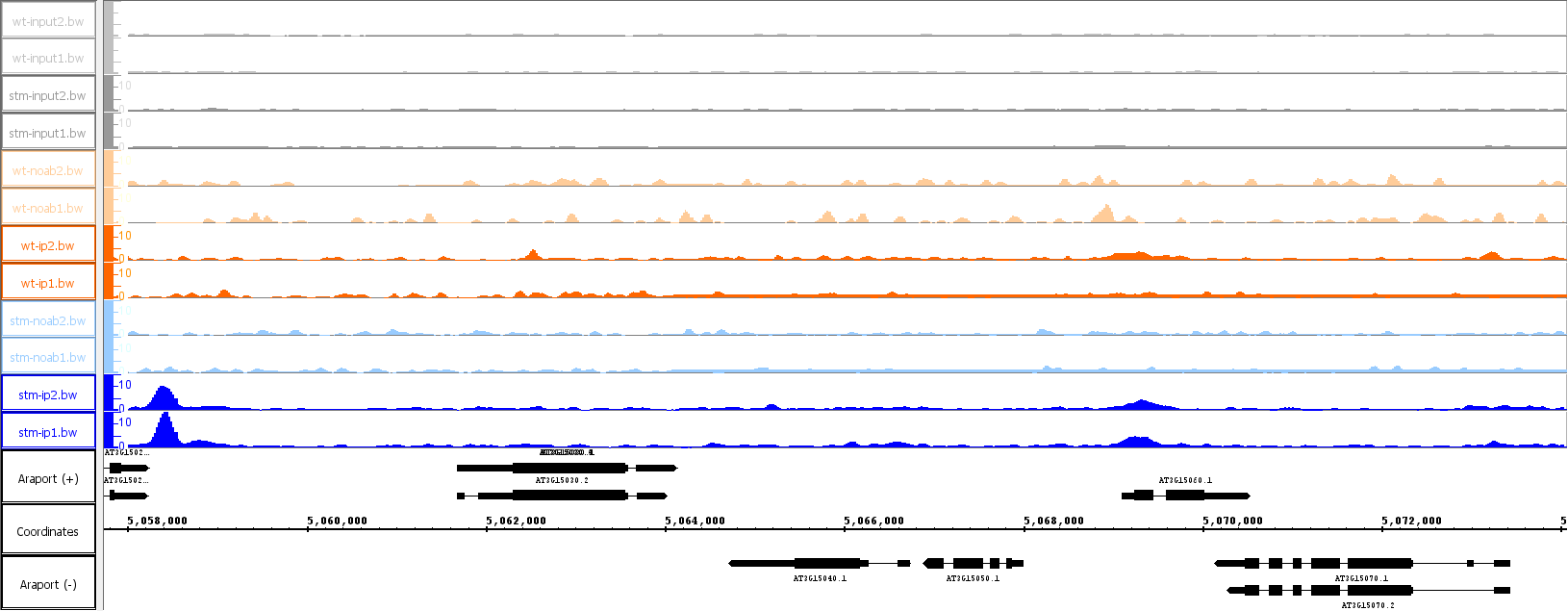


GRF7


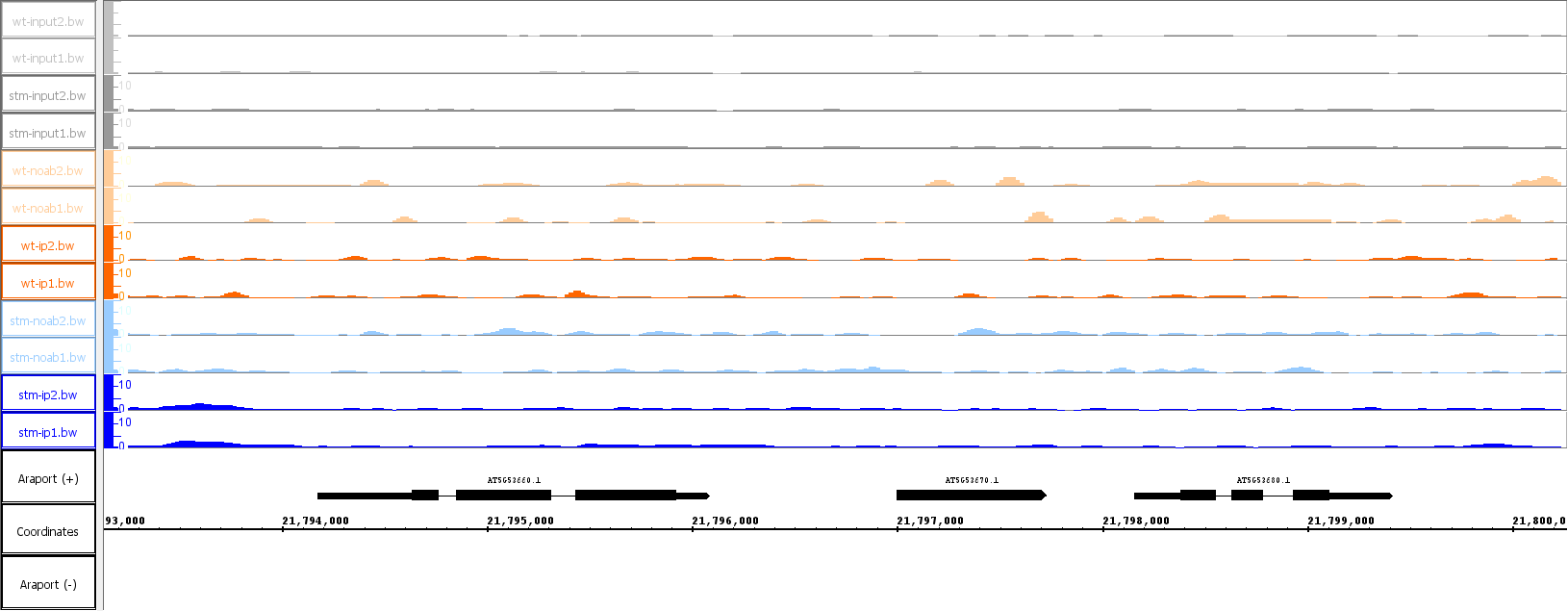


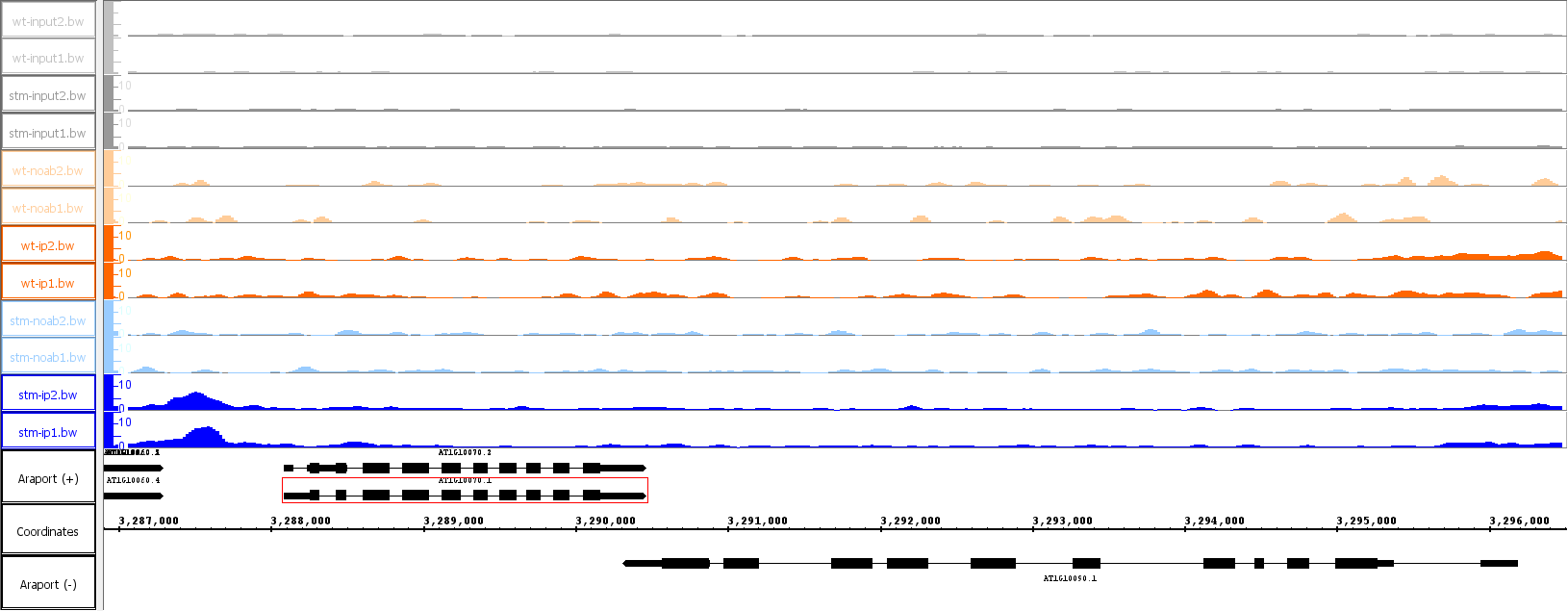


B-CAT2

CLV3


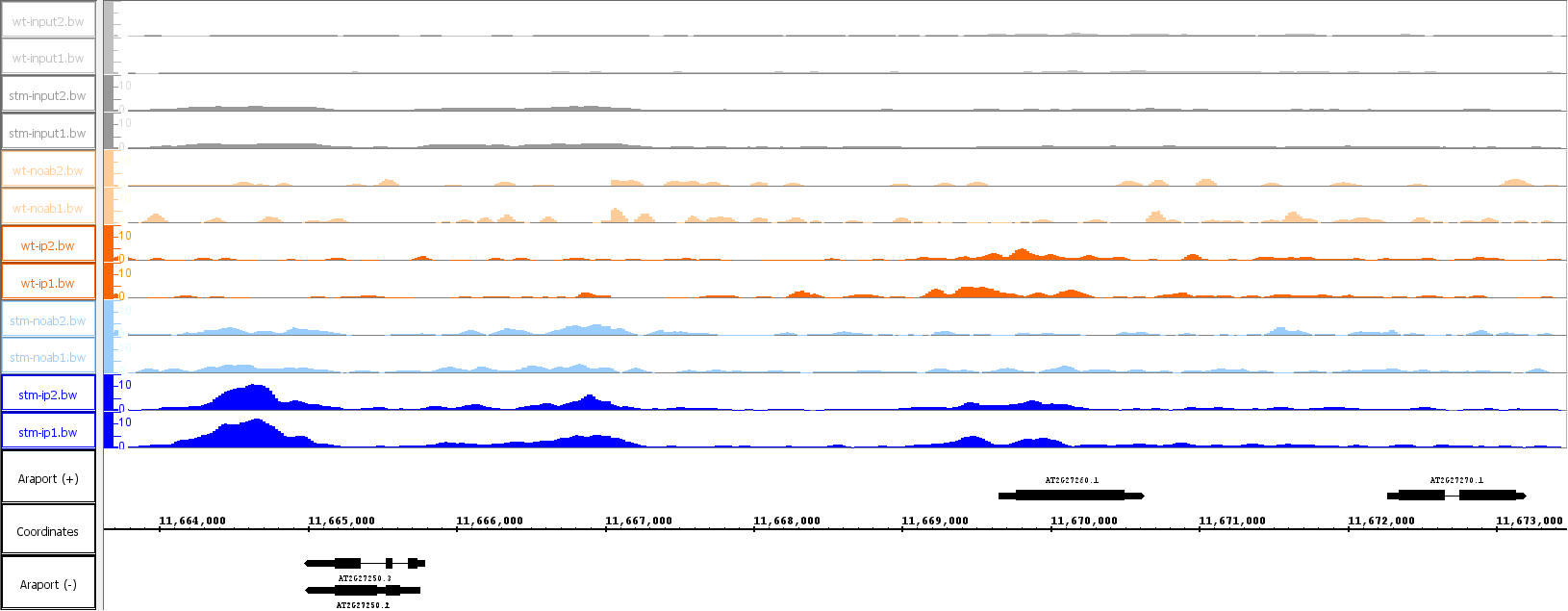


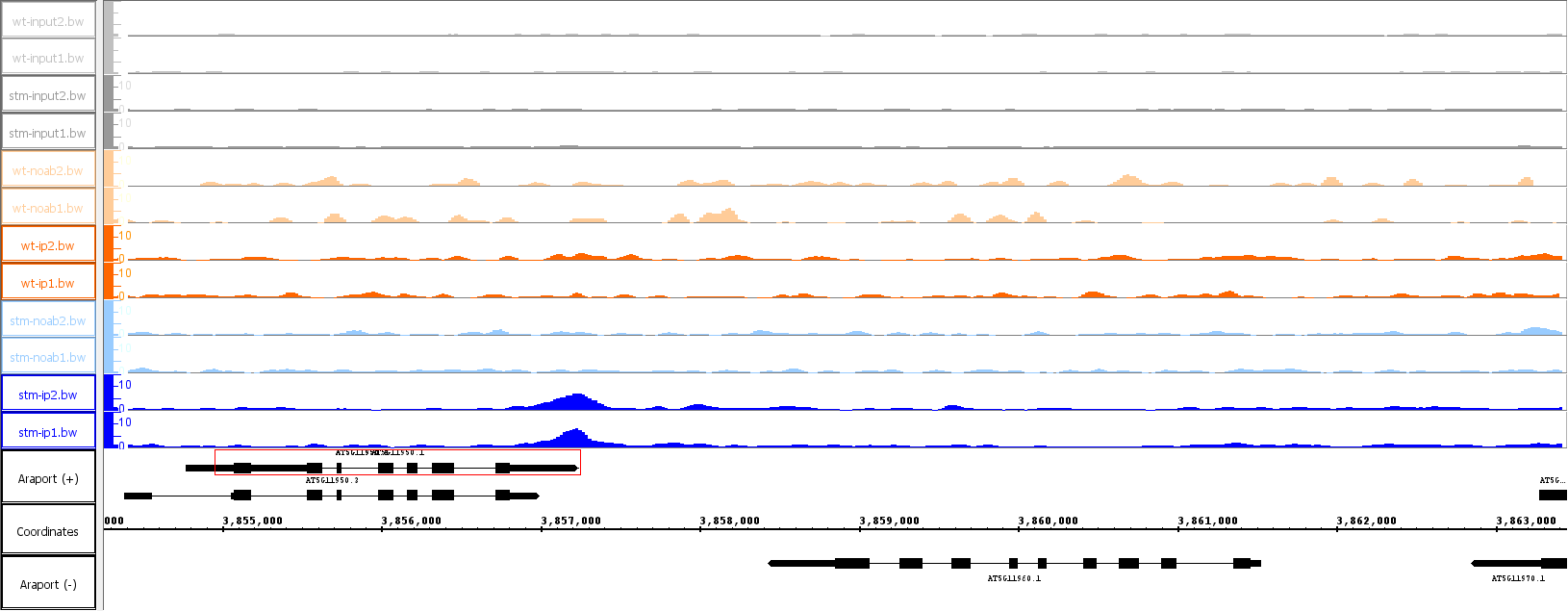


LOG8


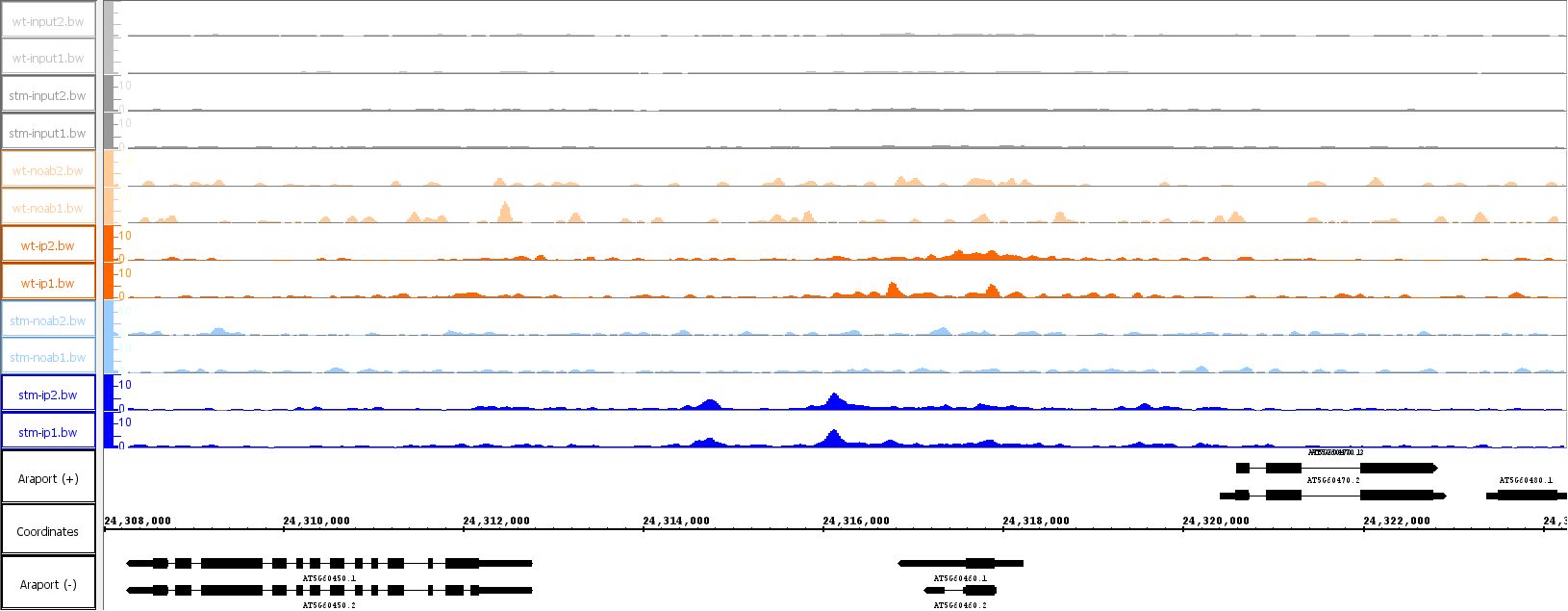


ARF4

MES9


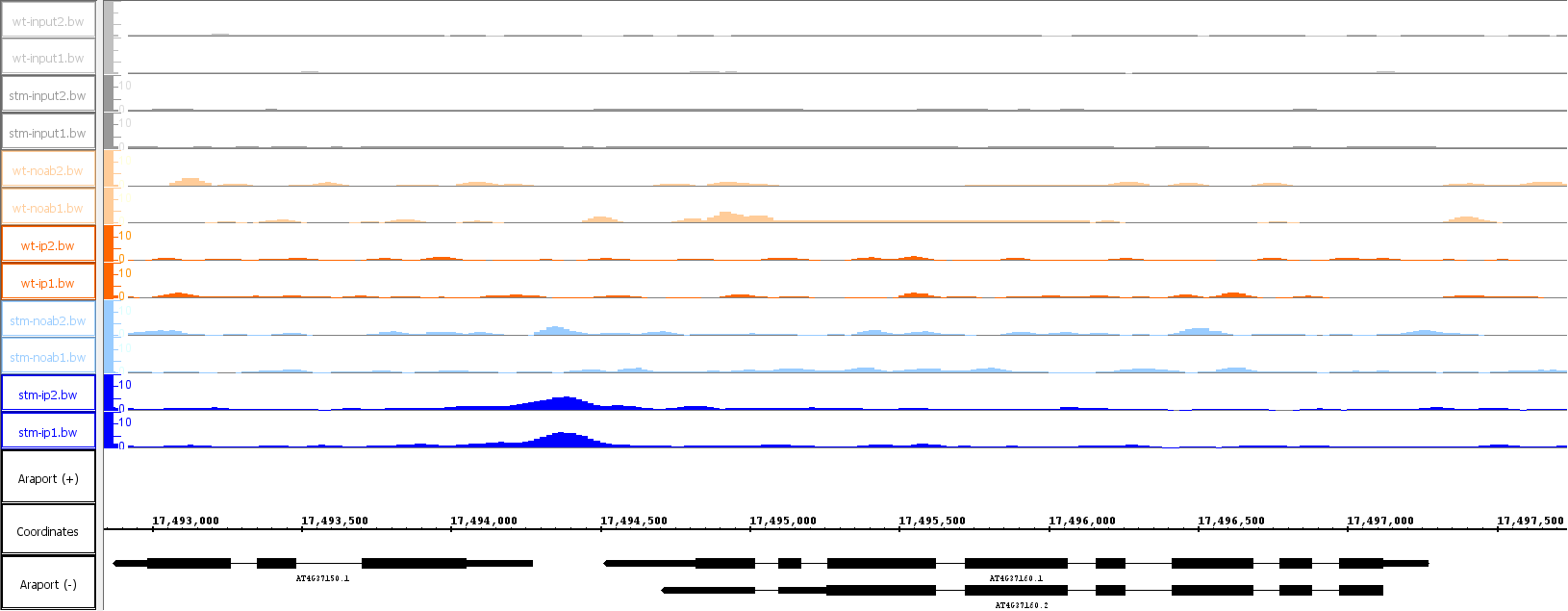


At3g24450


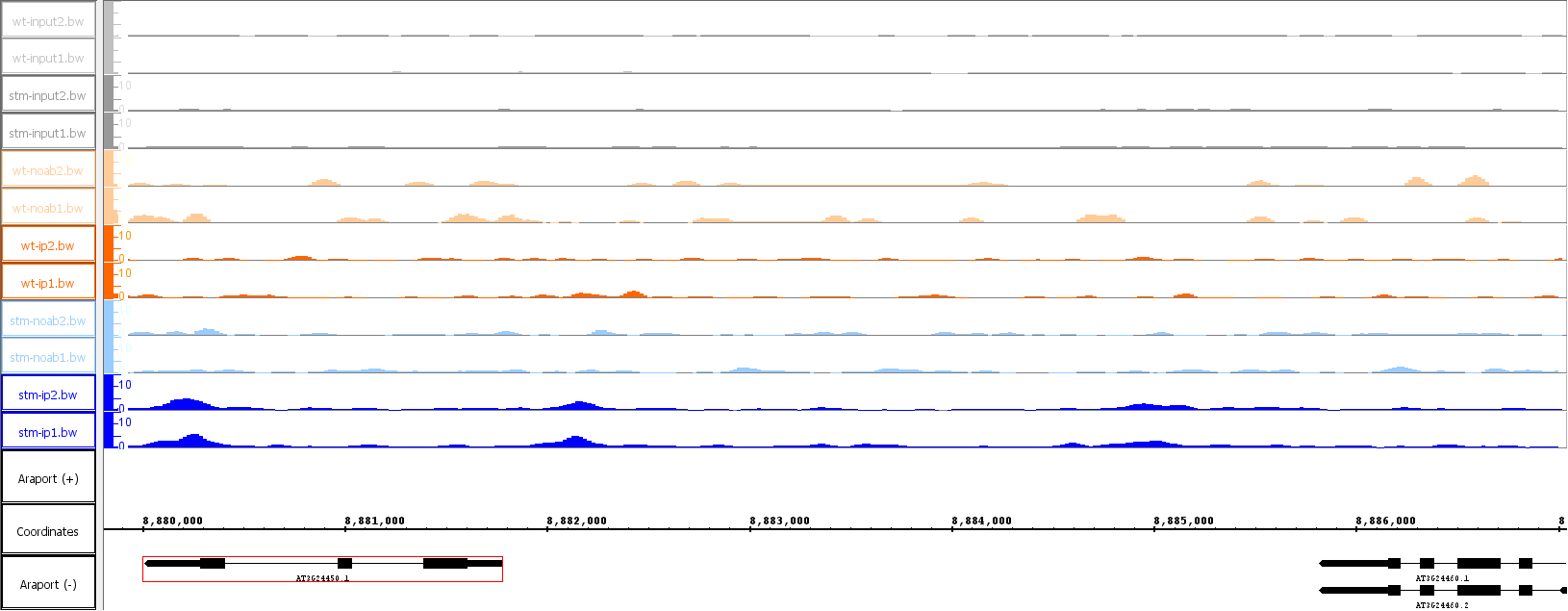


**Supplementary Figure S1. ChIP-seq peak data for selected STM target genes.** The STM immunoprecipitated samples (STM-IP1.bw and STM-IP2.bw) are highlighted in dark blue. The STM mock-immunoprecipitated (no-antibody) samples (STM-noab1.bw and STM-noab2.bw) are highlighted in light blue. The WT immunoprecipitated samples (WT-IP1.bw and WT-IP2.bw) are highlighted in orange. The WT mock-immunoprecipitated (no-antibody) samples (WT-noab1.bw and WT-noab2.bw) are highlighted in peach. The STM input (STM-input1.bw and STM-input2.bw) samples are shown in dark grey. The WT input (WT-input1.bw and WT-input2.bw) samples are shown in light grey. Peaks were visualised using the Integrated Genome Browser (IGB; Freese et al., 2016). Peaks detected in STM-IP samples, and indicated as peaks in Supplementary Table S1, are highlighted in red boxes. Lower tracks show gene models and genomic coordinates.


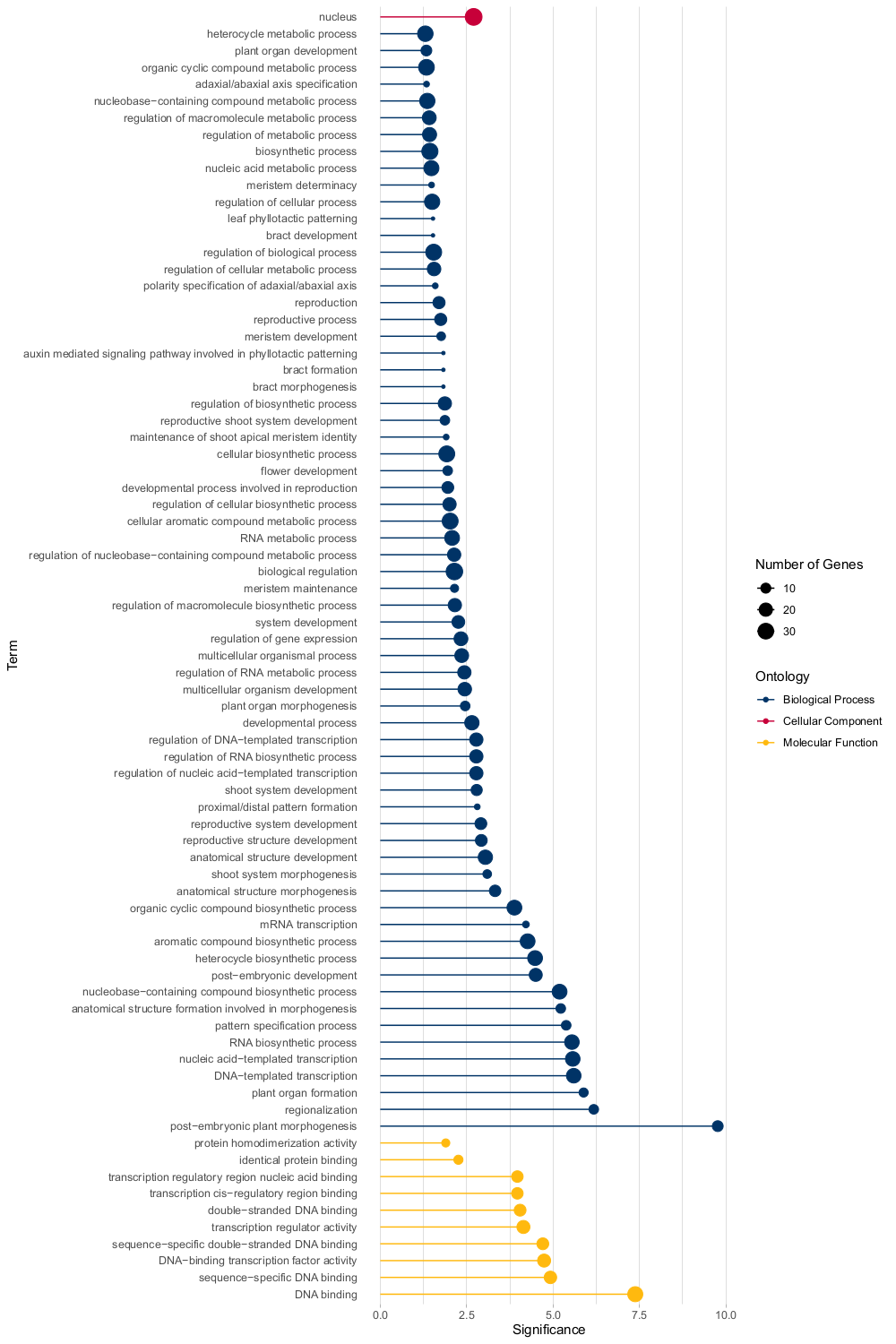


**Supplementary Figure S2. Gene Ontology enrichment analysis of ‘early responding’ STM target genes.** Terms related to development and hormones are highlighted in red boxes. Terms associated with transcriptional regulation are highlighted in blue boxes.


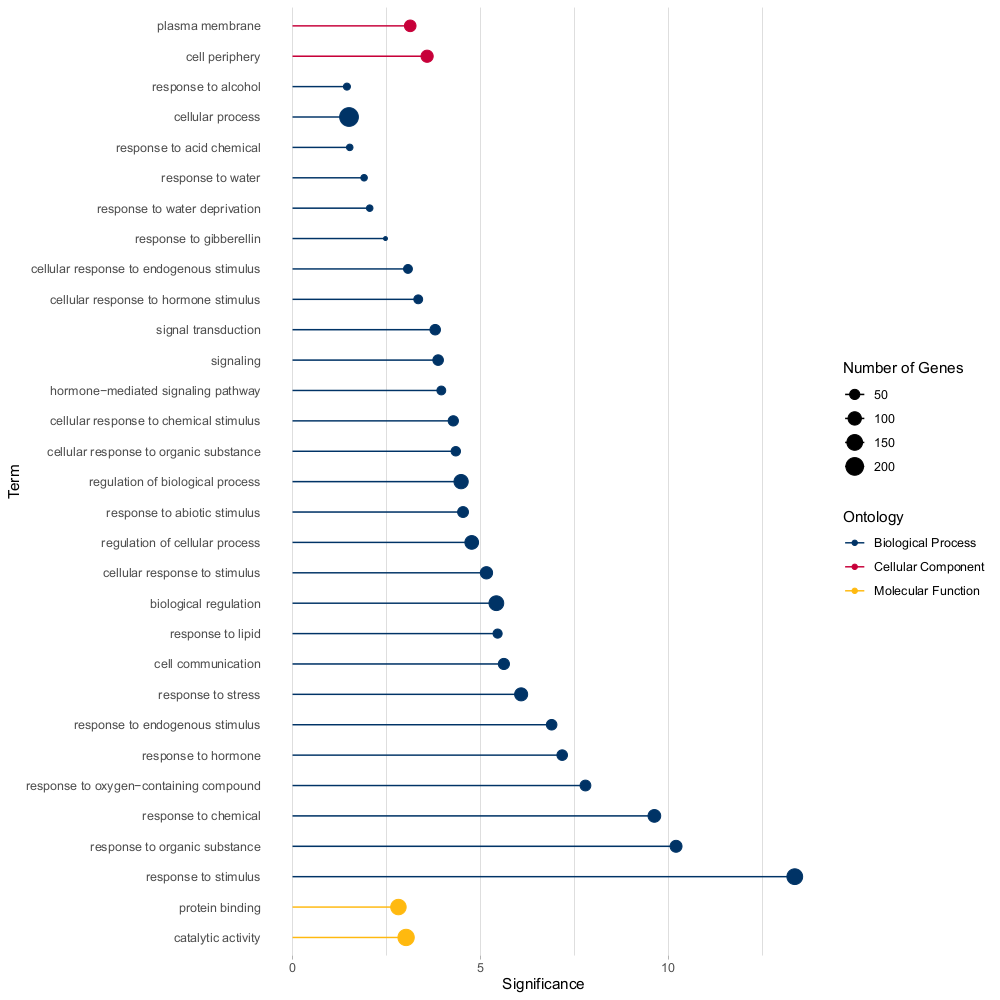


**Supplementary Figure S3. Gene Ontology enrichment analysis of ‘late responding’ STM target genes.** Terms related to development and hormones are highlighted in red boxes. Terms associated with transcriptional regulation are highlighted in blue boxes.


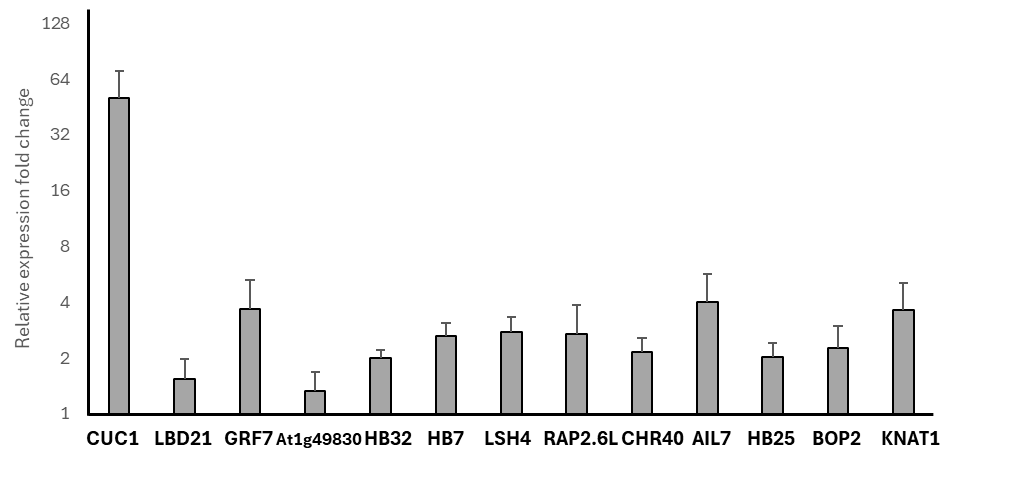


A

D

C

B


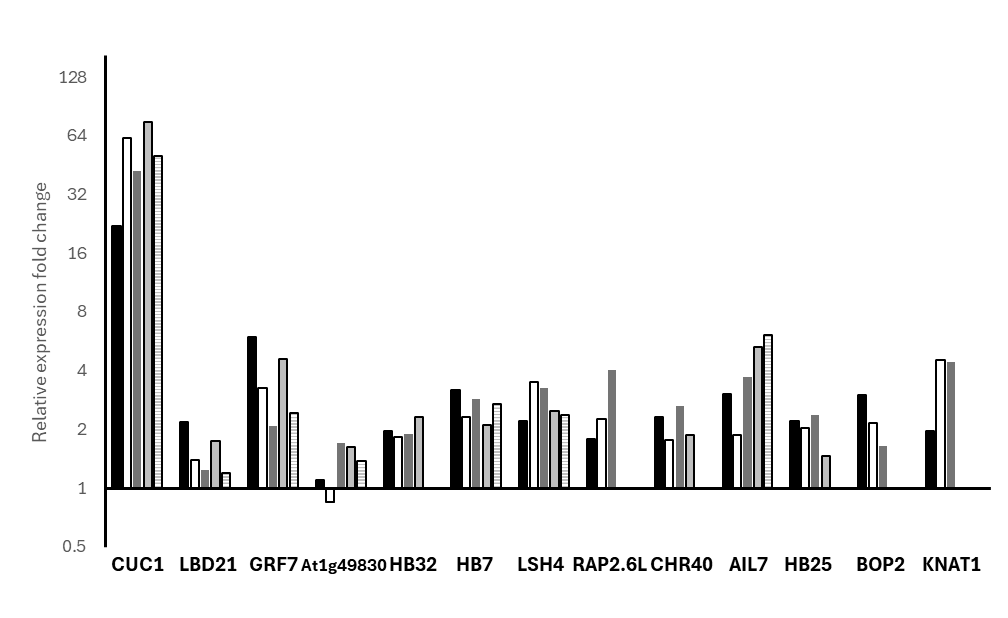


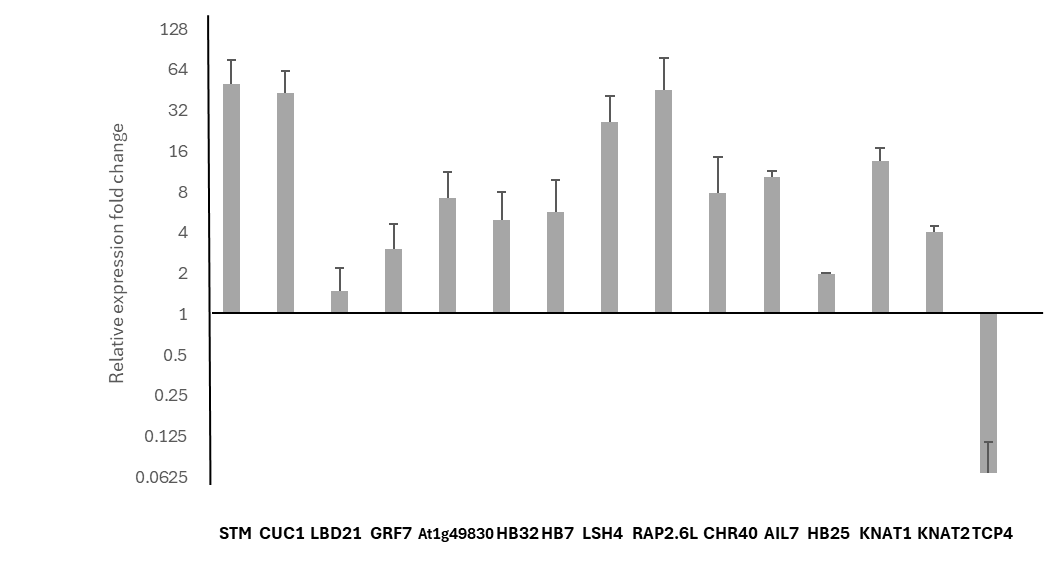


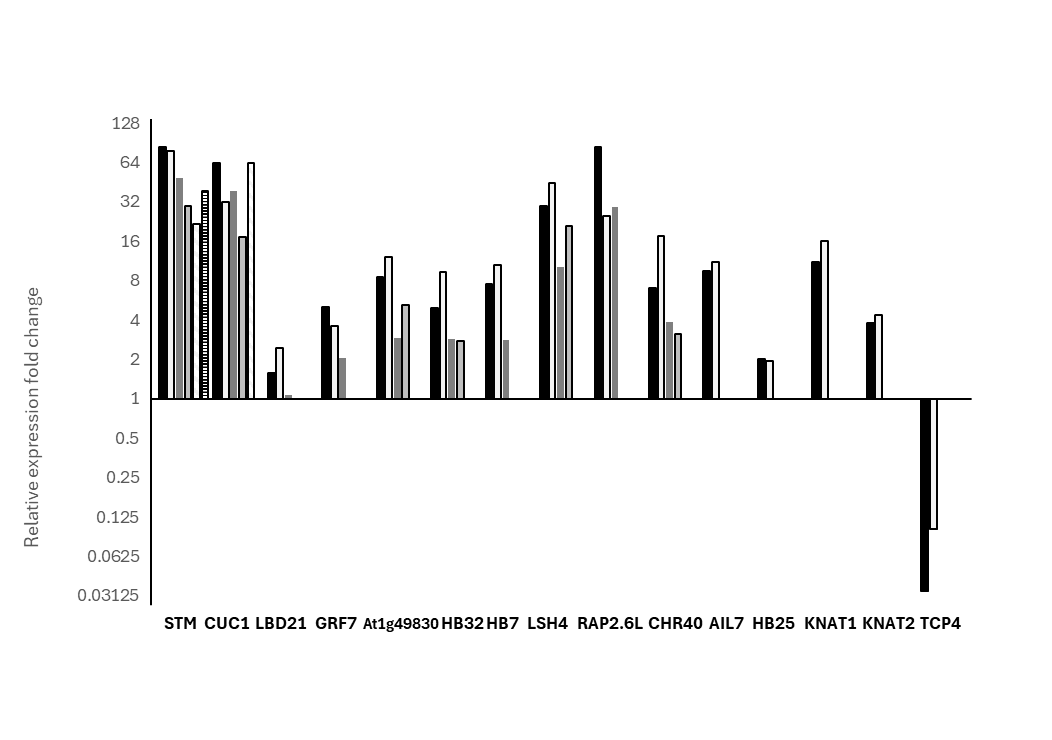


**Supplementary Figure S4.** qRT-PCR validation of a subset of putative STM target genes. (A) Average relative expression (fold change) of STM target genes 3h after induction with DEX in 9-11 day old 35S:STM-GR plants^20,29^. (B) Individual replicate data for STM target genes 3h after induction with DEX using the 35S:STM-GR line. Data are from multiple independent experiments, hence the different numbers of replicates for each gene. (C) Average relative expression (fold change) of STM target genes in 9-11 day old 35S:TGV;pTF:STM^29^ induced with DEX from germination. (D) Individual replicate data of STM target genes in 9-11 day old 35S:TGV;pTF:STM plants. Data are from multiple independent experiments, hence the different numbers of replicates for each gene. Error bars indicate standard deviations. All these genes showed statistical significant differential expression in original microarray experiment analyses using methods described in [29].
